# Supplementary material for: Association between childhood immunisation coverage and proximity to health facilities in rural settings: a cross-sectional analysis of Service Provision Assessment 2013–2014 facility data and Demographic and Health Survey 2015–2016 individual data in Malawi
Source: BMJ Open. 2022 Jul 25;12(7):e061346. doi: 10.1136/bmjopen-2022-061346 (PMC9328092; doi:10.1136/bmjopen-2022-061346)
Supplement: Supplementary data [file bmjopen-2022-061346supp001.pdf]

**Appendix Table 1. Additional study sample characteristics, children aged 12-23 months in rural areas, Malawi DHS 2015-16.**

| | Unweighted N | Weighted % |
|---|---|---|
| *Total* | *2740* | *100.0%* |
| **Individual child indicators** | | |
| Sex of child | | |
| Male | 1362 | 49.1% |
| Female | 1378 | 50.9% |
| Birth order | | |
| 1st | 673 | 25.3% |
| 2nd | 522 | 18.9% |
| 3rd | 453 | 16.1% |
| 4th | 375 | 13.2% |
| 5th or more | 717 | 26.5% |
| **Maternal/household indicators** | | |
| *Mother's age (Weighted Mean / SD)* | *27.2* | *6.6* |
| Mother's education | | |
| None | 331 | 13.2% |
| Primary | 1963 | 71.7% |
| Secondary or higher | 446 | 15.2% |
| Household wealth index quintile* | | |
| Poorest | 769 | 29.9% |
| Poorer | 678 | 24.8% |
| Middle | 601 | 22.3% |
| Richer | 464 | 16.1% |
| Richest | 228 | 7.0% |
| Region of Malawi | | |
| Northern | 508 | 12.0% |
| Central | 959 | 42.0% |
| Southern | 1273 | 46.0% |

**Appendix Table 2. Unadjusted and adjusted comparisons of immunization rates among children in rural areas aged 12-23 months by presence of vaccine-providing facility in 8km radius, Malawi 2013-14.**

| | **Unadjusted rates** | | | **Unadjusted logistic regression** | | **Adjusted logistic regression** | |
|---|---|---|---|---|---|---|---|
| | No vaccine-providing facility proximal | Vaccine-providing facility proximal | p-value | OR[1] | 95% CI | AOR[1,2] | 95% CI |
| *Specific immunization dose coverage* | | | | | | | |
| BCG | 96.4% | 97.8% | 0.22 | 1.59 | [0.71,3.58] | 1.54 | [0.70,3.39] |
| Rotavirus 2 doses | 87.0% | 91.9% | 0.02 | 1.66* | [1.03,2.65] | 1.53 | [0.95,2.45] |
| OPV 3 doses | 80.4% | 81.3% | 0.75 | 1.14 | [0.78,1.67] | 1.14 | [0.80,1.63] |
| Pentavalent 3 doses | 90.7% | 93.3% | 0.25 | 1.55 | [0.95,2.83] | 1.44 | [0.81,2.56] |
| Pneumococcal 3 doses | 87.2% | 89.4% | 0.54 | 1.33 | [0.67,2.64] | 1.20 | [0.64,2.26] |
| Measles 1+ dose | 86.0% | 92.0% | 0.03 | 2.04* | [1.16,3.60] | 1.88* | [1.06,3.33] |
| *Coverage of group of immunizations* | | | | | | | |
| All basic vaccines[3] | 69.1% | 76.6% | 0.08 | 1.57* | [1.02,2.43] | 1.54* | [1.02,2.32] |
| All recommended vaccines[4] | 36.7% | 53.2% | <0.001 | 1.89** | [1.28,2.81] | 1.97*** | [1.35,2.89] |
| *Negative immunization outcomes* | | | | | | | |
| Pentavalent zero dose | 5.4% | 2.3% | 0.03 | 0.37* | [0.16,0.88] | 0.38* | [0.18,0.82] |
| Pentavalent dropout[5] | 4.1% | 4.6% | 0.75 | 1.02 | [0.51,2.01] | 1.10 | [0.54,2.24] |

[1]Reference is children not proximal to a vaccine-providing facility
[2]Models also control for household wealth, mother's education, mother's age, child sex, child birth order, region of country
[3]Defined as BCG 1 dose, OPV 3 doses, DTP/HBV/Hib (pentavalent) 3 doses, measles 1 dose
[4]Basic + OPV birth dose + rotavirus 2 doses + PCV13 3 doses
[5]Dropout denominator is children with at least 1 dose of pentavalent
* p<0.05, ** p<0.01, *** p<0.001

**Appendix Table 3a. Logistic regression models of individual-level vaccination among children 12-23 months living in rural areas who are within 5km of a facility providing vaccination. Only facility-characteristic coefficients reported.[1]**

| | ***Specific immunization dose coverage*** | | | | | | | | | | | |
|---|---|---|---|---|---|---|---|---|---|---|---|---|
| | **BCG** | | **Rotavirus 2 dose** | | **Polio 3 dose** | | **Pentavalent 3 dose** | | **Pneumococcal 3 dose** | | **Measles 1+ dose** | |
| | AOR | 95% CI | AOR | 95% CI | AOR | 95% CI | AOR | 95% CI | AOR | 95% CI | AOR | 95% CI |
| *Facility type offering vaccination [highest available if >1]* | | | | | | | | | | | | |
| Hospital | *Ref* | *Ref* | *Ref* | *Ref* | *Ref* | *Ref* | *Ref* | *Ref* | *Ref* | *Ref* | *Ref* | *Ref* |
| Health centre | 0.92 | [0.34,2.49] | 1.03 | [0.54,1.95] | 1.07 | [0.72,1.58] | 0.89 | [0.48,1.64] | 0.69 | [0.40,1.20] | 1.17 | [0.51,2.64] |
| Health post/clinic/dispensary | 1.88 | [0.42,8.48] | 0.93 | [0.38,2.28] | 1.94 | [0.97,3.86] | 0.84 | [0.33,2.16] | 0.87 | [0.34,2.24] | 0.79 | [0.27,2.34] |
| *Managing authority of facility/ies offering vaccination* | | | | | | | | | | | | |
| Government | | | | | | | | | | | | |
| No | *Ref* | *Ref* | *Ref* | *Ref* | *Ref* | *Ref* | *Ref* | *Ref* | *Ref* | *Ref* | *Ref* | *Ref* |
| Yes | 2.06 | [0.51,8.36] | 1.65 | [0.89,3.07] | 2.32** | [1.37,3.93] | 1.76 | [0.91,3.42] | 1.18 | [0.64,2.15] | 1.34 | [0.70,2.55] |
| CHAM | | | | | | | | | | | | |
| No | *Ref* | *Ref* | *Ref* | *Ref* | *Ref* | *Ref* | *Ref* | *Ref* | *Ref* | *Ref* | *Ref* | *Ref* |
| Yes | 1.71 | [0.44,6.67] | 1.54 | [0.80,2.97] | 2.47*** | [1.46,4.18] | 2.04* | [1.06,3.93] | 1.05 | [0.60,1.86] | 1.39 | [0.63,3.07] |
| Private for-profit | | | | | | | | | | | | |
| No | *Ref* | *Ref* | *Ref* | *Ref* | *Ref* | *Ref* | *Ref* | *Ref* | *Ref* | *Ref* | *Ref* | *Ref* |
| Yes | 2.85 | [0.31,26.02] | 9.83* | [1.20,80.47] | 0.82 | [0.38,1.79] | 5.36 | [0.95,30.14] | 1.96 | [0.33,11.49] | 1.57 | [0.32,7.73] |
| NGO | | | | | | | | | | | | |
| No | *Ref* | *Ref* | *Ref* | *Ref* | *Ref* | *Ref* | *Ref* | *Ref* | *Ref* | *Ref* | *Ref* | *Ref* |
| Yes | 0.53 | [0.13,2.18] | 1.42 | [0.42,4.81] | 0.57 | [0.26,1.28] | 2.14 | [0.54,8.44] | 0.76 | [0.27,2.14] | 1.59 | [0.46,5.48] |

[1]Models also control for household wealth, education, mother's age, child sex, child birth order, region of country
* p<0.05, ** p<0.01, *** p<0.001

**Appendix Table 3b. Logistic regression models of individual-level vaccination among children 12-23 months living in rural areas who are within 5km of a facility providing vaccination. Only facility-characteristic coefficients reported.[1]**

| | ***Coverage of group of immunizations*** | | | | ***Negative immunization outcomes*** | | | |
|---|---|---|---|---|---|---|---|---|
| | **All basic vaccines[2]** | | **All recommended vaccines[3]** | | **Pentavalent 0 dose** | | **Pentavalent dropout** | |
| | AOR | 95% CI | AOR | 95% CI | AOR | 95% CI | AOR | 95% CI |
| *Facility type offering vaccination [highest available if >1]* | | | | | | | | |
| Hospital | *Ref* | *Ref* | *Ref* | *Ref* | *Ref* | *Ref* | *Ref* | *Ref* |
| Health centre | 1.11 | [0.75,1.66] | 0.79 | [0.55,1.14] | 1.46 | [0.42,5.12] | 0.95 | [0.49,1.86] |
| Health post/clinic/dispensary | 1.45 | [0.75,2.81] | 0.93 | [0.56,1.54] | 0.20 | [0.03,1.28] | 1.53 | [0.57,4.09] |
| *Managing authority of facility/ies offering vaccination* | | | | | | | | |
| Government | | | | | | | | |
| No | *Ref* | *Ref* | *Ref* | *Ref* | *Ref* | *Ref* | *Ref* | *Ref* |
| Yes | 1.82* | [1.15,2.88] | 1.94** | [1.24,3.03] | 0.08*** | [0.02,0.33] | 0.97 | [0.44,2.11] |
| CHAM | | | | | | | | |
| No | *Ref* | *Ref* | *Ref* | *Ref* | *Ref* | *Ref* | *Ref* | *Ref* |
| Yes | 2.29*** | [1.44,3.63] | 1.33 | [0.86,2.07] | 0.11** | [0.02,0.50] | 0.64 | [0.31,1.32] |
| Private for-profit | | | | | | | | |
| No | *Ref* | *Ref* | *Ref* | *Ref* | -- | -- | *Ref* | *Ref* |
| Yes | 0.84 | [0.39,1.78] | 1.04 | [0.48,2.25] | -- | -- | 0.26 | [0.04,1.54] |
| NGO | | | | | | | | |
| No | *Ref* | *Ref* | *Ref* | *Ref* | -- | -- | *Ref* | *Ref* |
| Yes | 0.60 | [0.28,1.28] | 0.62 | [0.35,1.10] | -- | -- | 0.60 | [0.15,2.32] |

[1]Models also control for household wealth, education, mother's age, child sex, child birth order, region of country
[2]Defined as BCG 1 dose, OPV 3 dose, DTP/HBV/Hib (pentavalent) 3 dose, measles 1 dose
[3]Basic + OPV birth dose + rotavirus 2 doses + PCV13 3 doses
* p<0.05, ** p<0.01, *** p<0.001
