# Supplementary material for: Association between childhood immunisation coverage and proximity to health facilities in rural settings: a cross-sectional analysis of Service Provision Assessment 2013–2014 facility data and Demographic and Health Survey 2015–2016 individual data in Malawi
Source: BMJ Open. 2022 Jul 25;12(7):e061346. doi: 10.1136/bmjopen-2022-061346 (PMC9328092; doi:10.1136/bmjopen-2022-061346)
Supplement: Author's manuscript [file bmjopen-2022-061346.draft_revisions.pdf]

BMJ Open is committed to open peer review. As part of this commitment we make the peer review history of every article we publish publicly available.

When an article is published we post the peer reviewers' comments and the authors' responses online. We also post the versions of the paper that were used during peer review. These are the versions that the peer review comments apply to.

The versions of the paper that follow are the versions that were submitted during the peer review process. They are not the versions of record or the final published versions. They should not be cited or distributed as the published version of this manuscript.

BMJ Open is an open access journal and the full, final, typeset and author-corrected version of record of the manuscript is available on our site with no access controls, subscription charges or pay-per-view fees (http://bmjopen.bmj.com).

If you have any questions on BMJ Open's open peer review process please email info.bmjopen@bmj.com

BMJ Open

The association between childhood immunization coverage and proximity to health facilities in rural areas: A cross-sectional analysis of SPA 2013-14 facility and DHS 2015-16 individual data in Malawi

Journal:	*BMJ Open*
Manuscript ID	bmjopen-2022-061346
Article Type:	Original research
Date Submitted by the Author:	01-Feb-2022
Complete List of Authors:	Johns, Nicole; World Health Organization, Department of Data and Analytics<br>Hosseinpoor, Ahmad Reza; World Health Organization, Department of Data and Analytics<br>Chisema, Mike; Malawi Ministry of Health<br>Danovaro-Holliday, M Carolina; World Health Organization, Department of Immunization, Vaccines and Biologicals<br>Kirkby, Katherine; World Health Organization, Department of Data and Analytics<br>Schlotheuber, Anne; World Health Organization, Department of Data and Analytics<br>Shibeshi, Messeret; World Health Organization, Inter-Country Support Team for East and Southern Africa<br>Sodha, Samir V.; World Health Organization, Department of Immunization, Vaccines and Biologicals<br>Zimba, Boston; World Health Organization, Malawi Country Office
Keywords:	PUBLIC HEALTH, Community child health < PAEDIATRICS, Paediatric infectious disease & immunisation < PAEDIATRICS

SCHOLARONE™
Manuscripts

I, the Submitting Author has the right to grant and does grant on behalf of all authors of the Work (as defined in the below author licence), an exclusive licence and/or a non-exclusive licence for contributions from authors who are: i) UK Crown employees; ii) where BMJ has agreed a CC-BY licence shall apply, and/or iii) in accordance with the terms applicable for US Federal Government officers or employees acting as part of their official duties; on a worldwide, perpetual, irrevocable, royalty-free basis to BMJ Publishing Group Ltd ("BMJ") its licensees and where the relevant Journal is co-owned by BMJ to the co-owners of the Journal, to publish the Work in this journal and any other BMJ products and to exploit all rights, as set out in our licence.

The Submitting Author accepts and understands that any supply made under these terms is made by BMJ to the Submitting Author unless you are acting as an employee on behalf of your employer or a postgraduate student of an affiliated institution which is paying any applicable article publishing charge ("APC") for Open Access articles. Where the Submitting Author wishes to make the Work available on an Open Access basis (and intends to pay the relevant APC), the terms of reuse of such Open Access shall be governed by a Creative Commons licence – details of these licences and which Creative Commons licence will apply to this Work are set out in our licence referred to above.

Other than as permitted in any relevant BMJ Author's Self Archiving Policies, I confirm this Work has not been accepted for publication elsewhere, is not being considered for publication elsewhere and does not duplicate material already published. I confirm all authors consent to publication of this Work and authorise the granting of this licence.

For peer review only

The association between childhood immunization coverage and proximity to health facilities in rural areas: A cross-sectional analysis of SPA 2013-14 facility and DHS 2015-16 individual data in Malawi

Nicole E. Johns[1], Ahmad Reza Hosseinpoor[1*], Mike Chisema[2], M. Carolina Danovaro-Holliday[3], Katherine Kirkby,[1] Anne Schlotheuber,[1] Messeret Shibeshi,[4] Samir V. Sodha,[3] Boston Zimba[5]

[1]Department of Data and Analytics, World Health Organization, Geneva, Switzerland.
[2]Malawi Ministry of Health, Lilongwe, Malawi.
[3]Department of Immunization, Vaccines and Biologicals, World Health Organization, Geneva, Switzerland.
[4]Inter-Country Support Team for East and Southern Africa, World Health Organization, Harare, Zimbabwe
[5]Malawi Country Office, World Health Organization, Lilongwe, Malawi
*Corresponding author

Corresponding Author:
Ahmad Reza Hosseinpoor
hosseinpoora@who.int
Department of Data and Analytics
Division of Data, Analytics and Delivery for Impact
World Health Organization
20, Avenue Appia, CH-1211 Geneva 27, Switzerland

Manuscript word count: 3481

Key words: Childhood immunization; vaccination; equity; rural; Malawi

For peer review only

ABSTRACT

Objectives: Despite significant progress in childhood vaccination coverage globally, substantial inequality remains. Remote rural populations are increasingly recognized as a priority group for immunization service equity. Access for remote rural populations is driven by health system and individual factors; analyses examining both health facility and individual data offer insight into immunization equity.

Design & setting: Retrospective cross-sectional analysis of facility data from the 2013-14 Malawi Service Provision Assessment and individual data from the 2015-16 Malawi Demographic and Health Survey, linking children to facilities within a 5-kilometer radius. We examined associations between proximity to health facilities and vaccination receipt via bivariate comparisons and logistic regression models.

Participants: 2740 children aged 12-23 months living in rural areas.

Outcome measures: Immunization coverage for the six vaccines included in the Malawi EPI schedule for children under one at time of study, as well as two composite vaccination indicators, zero-dose pentavalent coverage, and pentavalent dropout.

Findings: 72% (706/977) of facilities offered childhood vaccination services. Among children in rural areas, 61% were proximal to (within 5km) a vaccine-providing facility. Proximity to a vaccine-providing health facility was associated with increased likelihood of having received the rotavirus vaccine (93% vs 88%, p=0.004) and measles vaccine (93% vs 89%, p=0.01) in bivariate tests. In adjusted comparisons, how close a child was to a health facility remained meaningfully associated with how likely they were to have received rotavirus vaccine (AOR 1.63, 95% CI 1.12-2.33) and measles vaccine (AOR 1.62, 95% CI 1.11-2.37).

Conclusion: Proximity to health facilities was significantly associated with likelihood of receipt for some, but not all, vaccines. Our findings reiterate the vulnerability of children residing far from static vaccination services; efforts that specifically target remote rural populations living far from health facilities are warranted to ensure equitable vaccination coverage.

For peer review only

STRENGTHS AND LIMITATIONS OF THE STUDY

- Health facility and individual data can be linked to better understand barriers to and enablers of health service access and equity.
- This study is the first to utilize a nationally-representative sample to examine distance to immunization services empirically.
- Euclidean distance to nearest services is straightforward, but does not reflect actual access, which is tied to road networks, geography, seasonality, vehicle access, and preferred facilities.
- Distance is only one of many factors which render a population hard-to-reach or hard-to-vaccinate.
- Distance from facilities is a simple and clear potential target for reducing health inequality, particularly for rural populations.

For peer review only

INTRODUCTION

Despite significant progress in childhood vaccination coverage globally since the establishment of the Expanded Programme on Immunization (EPI) in 1974, substantial inequality in vaccination coverage remains.[1-4] Within countries, immunization coverage tends to be lowest among the most disadvantaged subgroups, including those living in rural areas.[2 5 6] The Equity Reference Group for Immunization has identified remote rural populations as one of the most pressing priority areas of work for improving immunization equity.[7 8] The Immunization Agenda 2030 (IA2030) recognizes coverage and equity as strategic priorities and has the stated goal to leave no one behind.[9 10] Similarly, Gavi's most recent strategy centers leaving no one behind and indicates equity as an organizing principle and key goal.[11] Globally, governments and key stakeholders in immunization are recognizing equity, including reaching remote rural populations, as a central priority.[8]

Immunization services for remote rural populations involve a number of barriers. On the side of the procurement, distribution, and provision of vaccines, barriers include increased marginal costs of administration, challenges of sufficient health worker availability and training, physical access for supply delivery, and cold-chain continuity.[7] One of the largest barriers on the side of the remote rural patient to immunization provision is physical access to static or outreach services. Distance to a health facility is a commonly cited barrier to vaccination in nationally representative studies and systematic reviews.[12-15] Several studies have examined distance empirically, and find significant negative associations between distance to vaccination site and likelihood of vaccination, though all rely on subnational samples.[16-18] Distance to a health facility that routinely provides immunization services is likely associated with immunization coverage, particularly among rural populations.

The Malawi health system consists of public, private for profit, and private not for profit sectors; the majority of healthcare services are provided by the government, followed by the major religious provider Christian Health Association of Malawi (CHAM).[19] Though vaccination services may be available at all facilities, the majority of children receive vaccinations through government facilities or outreach clinics.[20] In the community, immunization services are most frequently provided by health workers known as health surveillance assistants (HSAs), who provide door-to-door visitations and staff village, outreach, and mobile clinics.[19 21] Routine immunization services are also supported by other periodic supplementary immunization activities (SIAs).[22-24]

As of the 2015-16 Malawi Demographic and Health Survey (DHS), an estimated 76% (95% CI 74%-78%) of children age 12-23 months had received all basic immunizations (defined as one dose of Bacillus Calmette-Guérin (BCG); three doses of oral polio vaccine (OPV); three doses of

pentavalent vaccine (diphtheria, tetanus, pertussis [DTP], hepatitis B virus [HBV], and *Haemophilus influenzae* type b [Hib]); and one dose of measles vaccine), a decline from 81% (95% CI 79-83%) in the 2010-11 DHS.[25] Contrary to findings in other sub-Saharan settings and prior research within Malawi, childhood vaccination coverage was in fact higher among children living in rural areas (77%, 95% CI 74%-79%) than children living in urban areas (70%, 95% CI 63%-76%).[12 25] However, such figures conceal inequalities in access to immunization *within* rural populations, where the most remote are still likely under-immunized.[7] Understanding inequities within rural populations is particularly relevant in Malawi, where 84% of the population live in rural areas.[26]

The policy of the Malawi Ministry of Health is that all Malawians should live within 8 kilometers (km) of a public health facility, though as of 2014 more than two million people were estimated to live further than this from care.[27] Several studies from Malawi suggest that distance to a facility providing vaccination services is a driver of inequitable immunization coverage for remote rural populations.[28 29] Research has documented the relationship between empirically-derived distance to facility and other healthcare services and outcomes in Malawi;[30-35] to our knowledge, no other study has examined distance to facilities providing vaccination services and immunization coverage in Malawi.

In this study, we hypothesize that children aged 12-23 months who are proximal to a vaccine-providing health facility will be significantly more likely to have received immunizations than children who live further than 5km from the nearest vaccine-providing facility. Substantial research has explored individual-level factors associated with vaccination, but less work has demonstrated an association between empirically-derived facility access and immunization coverage, particularly using nationally-representative data. The nature of the data available in Malawi pose a unique opportunity to explore this quantitatively.

METHODS

Data sources

Facility data come from the Malawi 2013-14 DHS Service Provision Assessment (SPA) survey, a census of formal-sector public and private health facilities in Malawi.[36] It includes all static sites but not outreach clinic locations. Facilities included in the survey were hospitals, health centres, dispensaries, clinics, and health posts; managing authorities included government, CHAM and other faith-based organizations, private sector for-profit organizations, non-governmental organizations (NGOs), and companies.

For peer review only - http://bmjopen.bmj.com/site/about/guidelines.xhtml

We used data from the Malawi 2015-16 DHS to determine immunization coverage among children aged 12-23 months. Vaccine doses included were those six in the Malawi EPI schedule for children under one at the time of data collection: BCG, OPV, pentavalent, PCV13, rotavirus vaccine, and measles-containing vaccine (Table 1).[37] Details regarding DHS sampling, implementation, and content are published elsewhere.[25] Individual, family, and community characteristics were also extracted from this dataset.

Table 1. Malawi EPI vaccination schedule for children under one year, 2015[1]

Age	Vaccine
Birth up to two weeks after birth	BCG<br>Birth dose OPV
6 weeks	OPV dose 1<br>Pentavalent dose 1<br>PCV13 dose 1<br>Rotavirus dose 1
10 weeks	OPV dose 2<br>Pentavalent dose 2<br>PCV13 dose 2<br>Rotavirus dose 2
14 weeks	OPV dose 3<br>Pentavalent dose 3<br>PCV13 dose 3
9 months	Measles dose 1

[1]Several changes to the EPI have gone into effect since 2015. The measles vaccine was replaced with measles and rubella vaccine in July 2017. The trivalent OPV was replaced with bivalent OPV in 2016, and inactivated polio vaccine (IPV) was introduced starting in December 2018. The malaria vaccine (RTS,S/ASO1) was also piloted in 2019 but is not yet in the national EPI vaccination schedule as of December2021.

For peer review only

Geographic data linkage

Facility geo-location was matched to individual data using the DHS-suggested technique of a Euclidian distance buffer around each DHS cluster centroid.[38] This buffer is 5km in rural areas; this corresponds with the DHS offset of cluster GPS locations by up to 5km in rural areas. Therefore, proximity to a vaccine-providing facility is defined in this analysis as children living in rural areas within 5km of a facility which indicated provision of vaccination services on-site and/or via outreach.

Measures

Facility data indicated vaccination provision, stock, fees, and preparedness. For the sake of these analyses, only presence of a health facility offering vaccination services was used. Neither fees nor an indicator of full EPI provision were used because very few facilities (2%) reported charging vaccination-specific fees, and most facilities offering any vaccination services offered all EPI vaccines (99% of facilities offering vaccines offered full EPI). Despite a 2-3 year gap between the facility survey (2013-14) and individual survey (2015-16, children born October 2013-February 2015), retroactive reporting of immunization for children 12-23 months reflects immunization receipt close to the time of facility survey, and provision of any vaccination services is likely to have remained constant over that time.

Immunization coverage was defined as the proportion of children aged 12–23 months who received the indicated vaccine dose. For OPV, pentavalent, and PCV13 vaccines, children were considered having received the complete series of each vaccine type if they had three doses. Birth dose OPV was considered separately from the three-dose OPV series. For rotavirus vaccine, two doses were considered the complete series, and for BCG and measles, one dose (or more) was considered complete. We also examined receipt of all basic vaccines, also termed full immunization with basic vaccine doses, (one dose BCG, three doses OPV, three doses pentavalent, and one dose measles) as well as receipt of all recommended vaccines (all basic vaccine doses plus birth dose OPV, two doses rotavirus, and three doses PCV13). Finally, we created indicators of zero-dose pentavalent coverage (no receipt of pentavalent vaccine) and pentavalent dropout (receipt of the first dose, but not the third dose, of pentavalent vaccine). For all vaccines, both mother's recall and vaccination card verification (with or without date) were used to determine coverage. The few 'Don't know' responses were coded as non-receipt of the corresponding vaccine/dose (<1% for all, N=0 to 10 per vaccine dose).

Analyses

All analyses were limited to rural populations, as defined by DHS.[39] We first used bivariate Pearson's chi-squared tests to assess associations between vaccination coverage and proximity to a vaccine-providing health facility among children living in rural areas, for all immunization outcomes. We then constructed unadjusted and adjusted logistic regression models to assess the association between proximity to a vaccine-providing health facility and immunization outcomes among children living in rural areas, with adjusted models controlling for: child's sex [male, female], birth order [1, 2, 3, 4, 5+], household wealth [quintile], mother's age [years], mother's education [none, primary, secondary or higher], and subnational region [Northern, Central, Southern]. All covariates were selected *a priori* based on established association with likelihood of immunization and availability in DHS survey data.[20 29 40]

We then constructed multinomial models to separate immunization verified by vaccine card and immunization noted via mother's recall, to examine whether the association between facility proximity and immunization was sensitive to the nature of vaccination ascertainment. We also modified to the proximity radius to 8km rather than the 5km suggested by DHS, to align with Malawi Ministry of Health policy and assess whether findings were sensitive to the distance radius used.[27 38] Finally, we restricted the sample to children residing within 5km of a vaccine-providing facility, and examined whether facility level of care and managing authority were associated with vaccination coverage in adjusted logistic regression models.

All individual-level analyses accounted for complex sampling design using provided DHS survey weights. Statistical significance was set at $p<0.05$ for Pearson's chi-squared tests, Adjusted Odds Ratios (AORs), and adjusted Relative Risk Ratios (aRRRs); 95% confidence intervals (CIs) are reported throughout. We conducted analyses using ArcGIS Pro 2.8.0 and STATA 15.1.

Ethics Approval

Ethical approval for data collection was obtained by the DHS Program and implementing partners at time of survey, participants provided informed consent at the time of data collection. This study was reviewed and approved by the National Health Sciences Research Committee of Malawi (#20220106).

Patient and public involvement

Due to the nature of this analysis, patients and the public were not involved in the design, conduct, reporting, or dissemination of this research.

only

RESULTS

Of 977 facilities surveyed, 72% (N=706) offered any childhood immunization services (Table 2).

Table 2. Health facilities offering childhood immunization services, Malawi SPA 2013-14.

	Unweighted N	Weighted %
Total	*706*	*100.0%*
Type of vaccination offerings		
% offering all EPI vaccines	696	98.6%
Facility type		
Central hospital	1	0.1%
District hospital	24	3.4%
Rural/community hospital	41	5.8%
Other hospital	31	4.4%
Health centre	465	65.9%
Maternity	3	0.4%
Dispensary	40	5.7%
Clinic	81	11.5%
Health post	20	2.8%
Managing authority		
Government/public	457	64.7%
Christian Health Association of Malawi (CHAM)	153	21.7%
Private for profit	39	5.5%
Other mission/faith-based	6	0.9%
Non-governmental organization (NGO)	19	2.7%
Company	32	4.5%

For peer review

Individual survey data included 2740 children aged 12-23 months living in rural areas. The majority of children were proximal to health facilities: 64% were within 5km of *any* health facility and 61% were within 5km of a vaccine-providing facility (Table 3). Proximity was often limited to a single facility; only 5% were within 5km of two or more vaccine-providing facilities.

Overall, immunization coverage among children in rural areas was above 80% for all examined individual vaccines: 97.5% 1-dose BCG, 82.2% 3-dose OPV, 93.4% 3-dose pentavalent, 91.1% 2-dose rotavirus, 89.7% 3-dose PCV13, and 91.7% 1-dose measles (Table 3). However, full immunization coverage was lower: two-thirds (76.8%) of children had all basic vaccine doses and only half (50.4%) had all vaccines recommended for children under one. Few children received no doses of pentavalent vaccine (2.5%), and among children who started their pentavalent vaccine series, 4.2% did not complete it (indicating dropout).

For peer review only - http://bmjopen.bmj.com/site/about/guidelines.xhtml

Table 3. Study sample - children aged 12-23 months in rural areas, Malawi DHS 2015-16.

	Unweighted N	Weighted %
Total	*2740*	*100.0%*
**Facility proximity indicators**		
Facility proximity within 5km		
Vaccine-providing facility	978	36.3%
Non-vaccine-providing facility only	64	2.6%
No facility	1698	61.2%
Proximal to vaccine-providing facility, by type[1]		
Hospital	403	12.1%
Health centre	1264	47.2%
Health post/clinic/dispensary	289	10.9%
Proximal to vaccine-providing facility, by managing authority[1]		
Government/public	1305	46.7%
CHAM	501	16.9%
Private for profit	53	2.8%
NGO	74	2.8%
**Immunization coverage indicators**		
Specific immunization dose coverage		
BCG	2673	97.5%
Rotavirus 2 doses	2510	91.1%
OPV 3 doses	2273	82.2%
Pentavalent 3 doses	2559	93.4%
Pneumococcal 3 doses	2460	89.7%
Measles 1+ dose	2524	91.7%
Coverage of group of immunizations		
All basic vaccines[2]	2129	76.8%
All recommended vaccines[3]	1450	50.4%
Negative immunization outcomes		
Pentavalent zero dose	61	2.5%
Pentavalent dropout[4]	120	4.2%

[1]Children could be proximal to more than one vaccine-providing facility and thus more than one vaccine-providing facility type/managing authority

[2]Defined as BCG 1 dose, OPV 3 doses, DTP/HBV/Hib (pentavalent) 3 doses, measles 1 dose

[3]Basic + OPV birth dose + rotavirus 2 doses + PCV13 3 doses

[4]Dropout denominator is children with at least 1 dose of pentavalent; represents percent of children receiving first but not third dose of pentavalent.

For peer review only

Children living in rural areas who were proximal to a vaccine-providing facility were more likely to have some, but not all, vaccine doses in unadjusted bivariate tests when compared to children who were not in proximity to a vaccine-providing facility: rotavirus (93% vs 88%, p=0.004), measles (93% vs 89%, p=0.01), and full immunization with all basic vaccines (79% vs 74%, p=0.04) and with all recommended vaccines (54% vs 44%, p<0.001) (Table 4). No significant bivariate differences were observed for BCG, OPV, pentavalent, or PCV13 vaccine, nor pentavalent zero dose or dropout.

These relationships hold true in fully adjusted regression models (Table 4). Compared to children living greater than 5km from the nearest facility providing immunization services, children living proximal to a vaccine-providing facility had greater odds of receiving rotavirus (AOR 1.63, 95% CI 1.12-2.33) and measles (AOR 1.62, 95% CI 1.11-2.37) vaccines. They also had marginally greater odds of receiving all basic vaccines (AOR 1.28, 95% CI 0.99-1.65, p=0.057) and greater odds of receiving all recommended vaccines (AOR 1.38, 95% CI 1.09-1.74). They had somewhat lower odds of being zero-dose for pentavalent (AOR 0.53, 95% CI 0.28-1.01, p=0.052). No statistically significant associations were observed with BCG, OPV, pentavalent, or PCV13 receipt, nor pentavalent dropout.

For peer review only

Table 4. Unadjusted and adjusted comparisons of immunization rates among children in rural areas aged 12-23 months by presence of vaccine-providing facility in 5km radius, Malawi 2013-14.

	**Unadjusted rates**			**Unadjusted logistic regression**		**Adjusted logistic regression**		**Adjusted multinomial logistic regression, three-level recording-method-specific outcome**			
								Mother recall		Vaccine card	
	No vaccine-providing facility proximal	Vaccine-providing facility proximal	p-value	OR[1]	95% CI	AOR[1,2]	95% CI	aRRR[1,2]	95% CI	aRRR[1,2]	95% CI
Specific immunization dose coverage											
BCG	97.4%	97.6%	0.78	1.09	[0.58,2.05]	1.17	[0.63,2.20]	1.28	[0.67,2.42]	1.15	[0.61,2.16]
Rotavirus 2 doses	88.5%	92.8%	0.004	1.68**	[1.17,2.41]	1.63**	[1.13,2.33]	1.71*	[1.12,2.60]	1.72**	[1.18,2.51]
OPV 3 doses	81.4%	82.6%	0.55	1.09	[0.83,1.42]	1.08	[0.83,1.40]	1.15	[0.75,1.75]	1.11	[0.85,1.46]
Pentavalent 3 doses	92.6%	94.0%	0.30	1.24	[0.83,1.87]	1.23	[0.84,1.82]	1.33	[0.85,2.09]	1.20	[0.80,1.79]
Pneumococcal 3 doses	88.9%	90.2%	0.49	1.15	[0.78,1.68]	1.07	[0.75,1.53]	1.09	[0.70,1.68]	1.1	[0.76,1.59]
Measles 1+ dose	89.3%	93.3%	0.01	1.68**	[1.14,2.48]	1.62*	[1.11,2.37]	1.52*	[1.04,2.24]	1.59*	[1.07,2.35]
Coverage of group of immunizations											
All basic vaccines[3]	73.8%	78.7%	0.04	1.31*	[1.01,1.69]	1.28	[0.99,1.65]	--	--	--	--
All recommended vaccines[4]	45.0%	53.7%	<0.001	1.42**	[1.13,1.78]	1.38**	[1.09,1.74]	--	--	--	--
Negative immunization outcomes											
Pentavalent zero dose	3.4%	1.9%	0.08	0.55	[0.28,1.10]	0.53	[0.28,1.01]	--	--	--	--
Pentavalent dropout[5]	4.1%	4.2%	0.91	1.02	[0.67,1.56]	1.05	[0.69,1.61]	--	--	--	--

[1]Reference is children not proximal to a vaccine-providing facility
[2]Models also control for household wealth, mother's education, mother's age, child sex, child birth order, region of country
[3]Defined as BCG 1 dose, OPV 3 doses, DTP/HBV/Hib (pentavalent) 3 doses, measles 1 dose
[4]Basic + OPV birth dose + rotavirus 2 dose + PCV13 3 dose
[5]Dropout denominator is children with at least 1 dose of pentavalent
* p<0.05, ** p<0.01, *** p<0.001

For peer review only

The majority of children in rural areas (80%, 95% CI 78%-82%) had a vaccination card which was seen. Associations between proximity to a vaccine-providing facility and immunization coverage were similar for both mother's recall and vaccination card documented immunization in multinomial models (Table 4). The majority of children in rural areas (80%, 95% CI 78%-82%) had a vaccination card which was seen. Children were both more likely to have vaccination card-recorded rotavirus vaccination (AOR 1.71, 95% CI 1.12-2.60) or mother-recalled rotavirus vaccination (AOR 1.72, 95% CI 1.18-2.51) if they were proximal to a vaccine-providing facility. Children were also both more likely to have vaccination card recorded measles vaccination (AOR 1.59, 95% CI 1.07-2.35) or mother-recalled measles vaccination (AOR 1.52, 95% CI 1.04-2.24) if they were proximal to a vaccine-providing facility. No significant associations between proximity to vaccine-providing facility and immunization, whether ascertained through mother recall or vaccination card, were observed for BCG, OPV, pentavalent, or PCV13 vaccines.

When considering proximity as 8km rather than 5km, 87% of children were proximal to a vaccine-providing facility; associations with immunization coverage were similar to those with 5km proximity definition (Appendix Table 1). We observed significantly greater immunization coverage among children within 8km of a vaccine-providing facility for rotavirus (91.9% vs 87.0%, p=0.02), measles (92.0% vs 86.0%, p=0.03), and all recommended vaccines (53.2% vs 36.7%, p=0.001) in unadjusted comparisons. Additionally, zero-dose pentavalent receipt was significantly less common among children within 8km of a vaccine-providing facility compared to those not (2.3% vs 5.4%, p=0.03). Findings were similar in adjusted models: measles (AOR 1.88, 95% CI 1.06-3.33, p=0.03), all basic vaccines (AOR 1.54, 95% CI 1.02-2.32, p=0.04), all recommended vaccines (AOR 1.97, 95% CI 1.35-2.89, p<0.001), zero-dose pentavalent (AOR 0.38, 95% CI 0.18-0.83, p=0.01). Rotavirus vaccine did not have a significant association with 8km proximity to a vaccine-providing facility in adjusted models (AOR 1.53, 95% CI 0.95-2.45, p=0.08). As with 5km findings, no significant associations were observed for OPV, BCG, pentavalent, or PCV13 vaccines.

When limited to children within 5km of a vaccine-providing facility, the level of the facility was not associated with any of the immunization indicators (Appendix Tables 2a, 2b). Proximity to a government-run facility offering vaccination services was associated with greater odds of receipt of 3-dose OPV (AOR 2.32, 95% CI 1.37-3.93), all basic vaccines (AOR 1.82, 95% CI 1.15-2.88), and all recommended vaccines (AOR 1.94, 95% CI 1.24-3.03), and lower odds of zero-dose pentavalent (AOR 0.08, 95% CI 0.02-0.33). Proximity to a CHAM facility offering vaccination services was associated with greater odds of receipt of 3-dose OPV (AOR 2.47, 95% CI 1.46-4.18), 3-dose pentavalent (AOR 2.04, 95% CI 1.05-3.93), and all basic vaccines (AOR 2.29, 95% CI 1.44-3.63), and lower odds of zero-dose pentavalent (AOR 0.11, 95% CI 0.02-0.50).

DISCUSSION

Among children aged 12-23 months living in rural areas, proximity to a health facility providing vaccination services was associated with increased likelihood of rotavirus and measles vaccine receipt, and thus receipt of all recommended vaccines, and with decreased likelihood of zero-dose pentavalent vaccination. Even when accounting for known child, mother, family, and geographic determinants of immunization, how close a child was to a health facility was meaningfully associated with how likely they were to have received certain vaccines.

Proximity to a vaccine-providing health facility among rural children can be considered a proxy for remote ruralness: those who are far from a vaccine-providing facility are also likely to be far from a population center and far from services more generally. Therefore, these findings indicate that remote rural children in Malawi were likely inequitably under-vaccinated at the time of the 2015-16 DHS. This is an important equity consideration; despite higher immunization coverage amongst rural children than urban children overall, these findings reiterate that rural populations are not a monolith and that inequities are present beyond urban/rural differences. While geographic distance is just one of many factors which render a population hard-to-reach or hard-to-vaccinate, it is relatively easy to define and target, and is a meaningful correlate of coverage.[41] Equity-focused interventions and monitoring efforts should therefore use as granular geographic delineations as possible, with particular focus on those populations furthest from care.

We observed differential associations by type of immunization. The measles vaccine is delivered on its own several months after other infant vaccinations, and the requirement of a specific healthcare visit to obtain it may exacerbate the barrier of increased distance for accessing care. While rotavirus vaccine is offered simultaneously with other vaccinations, it was introduced to the Malawi EPI in October 2012 and outreach efforts may still have been in scale-up at the time the children under consideration were eligible for vaccination. It was introduced with strict age restrictions (first dose at 6-12 weeks, 4 weeks between doses, second dose no later than 16 weeks), which were not formally removed from the Malawi EPI until 2017.[42] The narrow time range for vaccination created by these limits may have made the vaccine harder to access for populations far from facilities. Conversely, the null findings for 3-dose OPV and 3-dose pentavalent vaccine add evidence to the success of the Malawi EPI in ensuring access to these vaccines among rural populations more broadly.

In analyses restricted to children living within 5km of vaccine-providing facilities, we observed no differences in immunization coverage by facility level of care, but we did find significant differences by facility managing authority. Similar odds of immunization regardless of facility level are unsurprising given that immunization services do not require highly specialized equipment or intensive provider training. Greater likelihood of immunization when the proximal facility was managed by the government or CHAM likely reflects the higher rate of outreach efforts performed by these authorities , the lack of fees for immunization at these facilities, and greater resource availability, training, and oversight more broadly.[43] Additional public-sector outreach efforts in areas where the only health facilities are run privately may thus be warranted.

Our finding that immunization coverage is inequitably lower among children further from health facilities is particularly relevant in light of the COVID-19 pandemic and its effects on childhood immunization services. Pandemic effects on childhood immunization services include service provision limitations such as suspension of outreach activities, disruption and suspension of in-facility services, disruption to vaccine and supply availability, shortages of available healthcare workers, and service utilization limitations including travel restrictions, concern for health and safety in seeking services, and lack of knowledge of service availability.[44 45] Mitigation efforts within Malawi and in other country contexts reduced the disruptions to routine immunization, resulting in only a 1-2% decline in coverage of vaccinations at the national level for 2020.[46-48] However, remote rural patients are most likely to have experienced these disruptions, given the reliance on outreach services or on travel to seek care. Strategies will be needed to ensure that missed children are caught-up for equitable immunization coverage.[49]

For peer review only

Limitations

These findings must be considered in light of several limitations. First, individual data does not indicate where vaccination services were rendered; children may have received immunization services from an alternate facility than the one most proximal to them (including via outreach, the locations of which were not assessed in the SPA). Thus, it would be inappropriate to suggest a causal relationship between the most proximal facility's immunization service availability and an individual child's immunization. Second, general service availability may not reflect actual service readiness and availability at the time of immunization receipt. Third, two planned sensitivity analyses were not possible: separately analyzing facilities offering within-facility and outreach services was not possible as 99% of children who were proximal to a vaccine-providing facility were proximal to one offering both in-facility and outreach services; examining specific vaccination availability was not possible because 99% of children who were proximal to a vaccine-providing facility were proximal to one offering all six examined vaccines. Finally, the use of buffer distances does not account for actual travel distance, geography, or time, nor does it account for seasonal differences in physical access (e.g. due to rains). Similarly, the use of DHS cluster centroids does not reflect the actual household geolocation. Statistically, this will bias findings toward the null as the buffer distance used will not be precise; any associations observed are likely underestimates of the true strength of association. Recent studies support the use of other methods such as theoretical catchment areas for more accurate facility linkage.[50] However, given the research question and the variable size of administrative and catchment areas, buffer distance was considered appropriate for these analyses.

CONCLUSION

Findings from this study align with previous work demonstrating a significant association between immunization coverage and distance to vaccine-providing facilities,[16-18] and expand on this by utilizing a nationally-representative sample with a focus on children living in rural areas. Remote rural populations have been identified as a key target for improving immunization equity, and these findings reiterate the vulnerability of children residing far from static vaccination services. Efforts that target remote rural populations living far from health facilities are warranted to ensure equitable vaccination coverage. These analyses also suggest that health facility-level data can and should be used for further analyses of inequalities in immunization.

For peer review only

Abbreviations

AOR: adjusted odds ratio; aRRR: adjusted relative risk ratio; BCG: *Bacillus Calmette-Guérin*; CHAM: Christian Health Association of Malawi; CI: confidence interval; DHS: Demographic and Health Survey; DTP: diphtheria, tetanus, pertussis vaccine; EPI: Expanded Programme on Immunization; GPS: global positioning system; HBV: hepatitis B virus; Hib: *Haemophilus influenzae* type b; KM: kilometer; NGO: non-governmental organization; OPV: oral polio vaccine; PCV13: 13-valent pneumococcal conjugate vaccine; SIA: supplementary immunization activity; SPA: Service Provision Assessment

Funding

Funding for this work was provided by Gavi, the Vaccine Alliance. The funding source had no role in the study design, analysis, interpretation of data, or decision to publish results.

Disclaimer

The authors alone are responsible for the views expressed in this article and they do not necessarily represent the views, decisions or policies of the institutions with which they are affiliated.

Data availability

Data for this study are publicly available upon request through the DHS Program. The data are owned by the Government of Malawi and are archived and managed by The DHS Program. Interested researchers must register at https://dhsprogram.com/data/ and can then request permission to download the SPA and DHS datasets.

Analytical code for results presented here is available upon reasonable request from the corresponding author.

Conflicts of interests

The authors have no conflicts of interest to disclose.

Contributors

AH led initial study conceptualization and provided oversight of analyses and writing. NJ generated specific hypotheses, led data analyses, and created initial manuscript draft. MC, MS, and BZ provided country-specific context, content, and feedback for interpretation of the data. CD-H and SS provided immunization context, content, and feedback for interpretation of the data. KK and AS provided health equity context, content, and feedback for interpretation of the data. All authors contributed to substantive review and revision of the final manuscript, provided final approval of the version to be published, and agree to be accountable for all aspects of the work.

For peer review only

REFERENCES

1. Keja K, Chan C, Hayden G, et al. Expanded programme on immunization. *World health statistics quarterly Rapport trimestriel de statistiques sanitaires mondiales* 1988;41(2):59-63.
2. State of inequality: childhood immunization. Geneva: World Health Organization, 2016.
3. Galles NC, Liu PY, Updike RL, et al. Measuring routine childhood vaccination coverage in 204 countries and territories, 1980–2019: a systematic analysis for the Global Burden of Disease Study 2020, Release 1. *The Lancet* 2021;398(10299):503-21.
4. Muhoza P, Danovaro-Holliday MC, Diallo MS, et al. Routine Vaccination Coverage - Worldwide, 2020. *Mmwr-Morbid Mortal W* 2021;70(43):1495-500.
5. Explorations of inequality: Childhood immunization. Geneva, Switzerland: World Health Organization, 2018.
6. Hosseinpoor AR, Bergen N, Schlotheuber A, et al. State of inequality in diphtheria-tetanus-pertussis immunisation coverage in low-income and middle-income countries: a multicountry study of household health surveys. *The Lancet Global Health* 2016;4(9):e617-e26.
7. Levine O, Lemango E, Befson J, et al. ERG Discussion Paper 08: Tackling inequities in immunization outcomes in remote rural contexts. 2018. https://sites.google.com/view/erg4immunisation/discussion-papers (accessed 09/21/21).
8. Chopra M, Bhutta Z, Blanc DC, et al. Addressing the persistent inequities in immunization coverage. *B World Health Organ* 2020;98(2):146-48. doi: 10.2471/Blt.19.241620
9. World Health Organization. Immunization Agenda 2030: a global strategy to leave no one behind. *Geneva: WHO* 2020
10. Lindstrand A, Cherian T, Chang-Blanc D, et al. The World of Immunization: Achievements, Challenges, and Strategic Vision for the Next Decade. *The Journal of infectious diseases* 2021;224(Supplement_4):S452-S67.
11. Gavi The Vaccine Alliance. Phase V (2021–2025) 2019 [updated 9 Jun 2021. Available from: https://www.gavi.org/our-alliance/strategy/phase-5-2021-2025.
12. Bangura JB, Xiao S, Qiu D, et al. Barriers to childhood immunization in sub-Saharan Africa: A systematic review. *BMC Public Health* 2020;20(1):1108. doi: 10.1186/s12889-020-09169-4 [published Online First: 2020/07/16]
13. Rahman M, Obaida-Nasrin S. Factors affecting acceptance of complete immunization coverage of children under five years in rural Bangladesh. *Salud Publica Mex* 2010;52(2):134-40. doi: 10.1590/s0036-36342010000200005 [published Online First: 2010/05/21]
14. Adedokun ST, Uthman OA, Adekanmbi VT, et al. Incomplete childhood immunization in Nigeria: a multilevel analysis of individual and contextual factors. *BMC Public Health* 2017;17(1):236. doi: 10.1186/s12889-017-4137-7 [published Online First: 2017/03/09]
15. Phillips DE, Dieleman JL, Lim SS, et al. Determinants of effective vaccine coverage in low and middle-income countries: a systematic review and interpretive synthesis. *Bmc Health Serv Res* 2017;17 doi: ARTN 681

10.1186/s12913-017-2626-0
16. Tefera YA, Wagner AL, Mekonen EB, et al. Predictors and Barriers to Full Vaccination among Children in Ethiopia. *Vaccines (Basel)* 2018;6(2) doi: 10.3390/vaccines6020022 [published Online First: 2018/04/13]
17. Miyahara R, Jasseh M, Gomez P, et al. Barriers to timely administration of birth dose vaccines in The Gambia, West Africa. *Vaccine* 2016;34(29):3335-41. doi: 10.1016/j.vaccine.2016.05.017 [published Online First: 2016/05/20]
18. Kiptoo E, Esilaba M, Kobia G, et al. Factors influencing low immunization coverage among children between 12-23 months in East Pokot, Baringo Country, Kenya. *Int J Vaccines* 2015;1(2):00012.
19. Ministry of Health. Financial Sustainability Plan (FSP) for Expanded Programme on Immunization. Linongwe, Malawi, 2004.
20. Munthali AC. Determinants of vaccination coverage in Malawi: evidence from the demographic and health surveys. *Malawi Med J* 2007;19(2):79-82. doi: 10.4314/mmj.v19i2.10934 [published Online First: 2007/06/01]
21. Nyirenda L, Flikke R. Frontline Vaccinators and Immunisation Coverage in Malawi. *Forum Dev Stud* 2013;40(1):27-46. doi: 10.1080/08039410.2012.725676
22. Minetti A, Kagoli M, Katsulukuta A, et al. Lessons and challenges for measles control from unexpected large outbreak, Malawi. *Emerg Infect Dis* 2013;19(2):202-9. doi: 10.3201/eid1902.120301 [published Online First: 2013/01/25]
23. Chinele J. Over a million children get protected against polio in Malawi. 2021 26 August 2021. https://www.gavi.org/vaccineswork/over-million-children-get-protected-against-polio-malawi (accessed 09/21/2021).

For peer review only

24. Kainga HW, Ssendagire S, Ssanyu JN, et al. Proportion of children aged 9-59 months reached by the 2017 measles supplementary immunization activity among the children with or without history of measles vaccination in Lilongwe district, Malawi. *PLoS One* 2021;16(1):e0243137. doi: 10.1371/journal.pone.0243137 [published Online First: 20210111]
25. National Statistical Office (NSO) [Malawi] and ICF. Malawi Demographic and Health Survey 2015-16. Zomba, Malawi, and Rockville, Maryland, USA.: NSO and ICF., 2017.
26. 2018 Population and Housing Census Main Report. Zomba, Malawi: Malawi National Statistical Office, 2019.
27. Ministry of Health. 2014/15 ANNUAL REVIEW REPORT FOR THE HEALTH SECTOR. Linongwe, Malawi, 2015.
28. Okeibunor JC, Ogbuanu I, Blanche A, et al. Towards a Strategy for Reducing Missed Opportunities for Vaccination in Malawi: Implications of a Qualitative Health Facility Assessment. *J Immunol Sci* 2018;Suppl(7):46-54. [published Online First: 2019/02/16]
29. Ntenda PAM, Nkoka O, Nana AW, et al. Factors associated with completion of childhood immunization in Malawi: a multilevel analysis of the 2015-16 Malawi demographic and health survey. *Trans R Soc Trop Med Hyg* 2019 doi: 10.1093/trstmh/trz029 [published Online First: 2019/04/30]
30. McGuire F, Kreif N, Smith PC. The effect of distance on maternal institutional delivery choice: Evidence from Malawi. *Health Econ* 2021;30(9):2144-67. doi: 10.1002/hec.4368 [published Online First: 2021/06/08]
31. Leslie HH, Fink G, Nsona H, et al. Obstetric Facility Quality and Newborn Mortality in Malawi: A Cross-Sectional Study. *PLoS Med* 2016;13(10):e1002151. doi: 10.1371/journal.pmed.1002151 [published Online First: 2016/10/19]
32. Peven K, Taylor C, Purssell E, et al. Distance to available services for newborns at facilities in Malawi: A secondary analysis of survey and health facility data. *PloS one* 2021;16(7):e0254083.
33. Mallick L, Benedict RK, Wang W. Facility readiness and counseling during antenatal care and the relationship with early breastfeeding in Haiti and Malawi. *BMC Pregnancy Childbirth* 2020;20(1):325. doi: 10.1186/s12884-020-02919-7 [published Online First: 2020/05/31]
34. Benedict R, Wang W, Mallick L. Examining the Role of Health Facilities in Supporting Iron Folic Acid Supplementation Adherence Among Women in Malawi (OR25-03-19). *Current developments in nutrition* 2019;3(Supplement_1):nzz051. OR25-03-19.
35. Digitale J, Psaki S, Soler-Hampejsek E, et al. Correlates of contraceptive use and health facility choice among young women in Malawi. *The ANNALS of the American Academy of Political and Social Science* 2017;669(1):93-124.
36. Ministry of Health - MoH/Malawi and ICF International. Malawi Service Provision Asessment (MSPA) 2013-14. Lilongwe, Malawi: MOH/Malawi and ICF International, 2014.
37. EPI Comprehensive Multi-Year Plan 2016-2020. 2015. https://extranet.who.int/countryplanningcycles/sites/default/files/planning_cycle_repository/malawi/malawi_cmyp_2016-2020.pdf (accessed 09/21/2021).
38. Burgert CR, Prosnitz D. Linking DHS Household and SPA Facility Surveys: Data Considerations and Geospatial Methods. DHS Spatial Analysis Reports No 10. Rockville, Maryland, USA: ICF, 2014.
39. Fish TD, Janocha B, Dontamsetti T, et al. GEOSPATIAL COVARIATES: PROXIES FOR MAPPING URBAN-RELATED INDICATORS. DHS SPATIAL ANALYSIS REPORTS 19. Rockville, Maryland, USA: ICF, 2020.
40. Wiysonge CS, Uthman OA, Ndumbe PM, et al. Individual and contextual factors associated with low childhood immunisation coverage in sub-Saharan Africa: a multilevel analysis. *PloS one* 2012;7(5):e37905.
41. Ozawa S, Yemeke TT, Evans DR, et al. Defining hard-to-reach populations for vaccination. *Vaccine* 2019;37(37):5525-34. doi: 10.1016/j.vaccine.2019.06.081
42. Mandomando I, Mumba M, Biey JNM, et al. Implementation of the World Health Organization recommendation on the use of rotavirus vaccine without age restriction by African countries. *Vaccine* 2021;39(23):3111-19. doi: 10.1016/j.vaccine.2021.03.021
43. Makwero MT. Delivery of primary health care in Malawi. *Afr J Prim Health Ca* 2018;10(1) doi: ARTN a1799

10.4102/phcfm.v10i1.1799
44. Olorunsaiye CZ, Yusuf KK, Reinhart K, et al. COVID-19 and Child Vaccination: A Systematic Approach to Closing the Immunization Gap. *International Journal of Maternal and Child Health and AIDS* 2020;9(3):381.
45. Shet A, Carr K, Danovaro-Holliday MC, et al. Impact of the SARS-CoV-2 pandemic on routine immunisation services: evidence of disruption and recovery from 170 countries and territories. *Lancet Glob Health* 2021 doi: 10.1016/S2214-109X(21)00512-X [published Online First: 20211221]

46. Dixit SM, Sarr M, Gueye DM, et al. Addressing disruptions in childhood routine immunisation services during the COVID-19 pandemic: perspectives from Nepal, Senegal and Liberia. *Bmj Glob Health* 2021;6(7) doi: ARTN e005031

10.1136/bmjgh-2021-005031

47. Chinele J. Tackling Malawi's fears of routine immunisation: "Children should still be immunised amid the pandemic": Gavi: The Vaccine Alliance; 2021 [updated 24 June 2021. Available from: https://www.gavi.org/vaccineswork/tackling-malawis-fears-routine-immunisation-children-should-still-be-immunised-amid.

48. World Health Organization, UNICEF. Malawi: WHO and UNICEF estimates of immunization coverage: 2020 revision, 2021.

49. World Health Organization. Leave no one behind : guidance for planning and implementing catch-up vaccination, 2021.

50. Peters MA, Mohan D, Naphini P, et al. Linking household surveys and facility assessments: a comparison of geospatial methods using nationally representative data from Malawi. *Population health metrics* 2020;18(1):1-11.

For peer review only

Appendix Table 1. Unadjusted and adjusted comparisons of immunization rates among children in rural areas aged 12-23 months by presence of vaccine-providing facility in 8km radius, Malawi 2013-14.

	Unadjusted rates			Unadjusted logistic regression		Adjusted logistic regression	
	No vaccine-providing facility proximal	Vaccine-providing facility proximal	p-value	OR[1]	95% CI	AOR[1,2]	95% CI
Specific immunization dose coverage							
BCG	96.4%	97.8%	0.22	1.59	[0.71,3.58]	1.54	[0.70,3.39]
Rotavirus 2 doses	87.0%	91.9%	0.02	1.66*	[1.03,2.65]	1.53	[0.95,2.45]
OPV 3 doses	80.4%	81.3%	0.75	1.14	[0.78,1.67]	1.14	[0.80,1.63]
Pentavalent 3 doses	90.7%	93.3%	0.25	1.55	[0.95,2.83]	1.44	[0.81,2.56]
Pneumococcal 3 doses	87.2%	89.4%	0.54	1.33	[0.67,2.64]	1.20	[0.64,2.26]
Measles 1+ dose	86.0%	92.0%	0.03	2.04*	[1.16,3.60]	1.88*	[1.06,3.33]
Coverage of group of immunizations							
All basic vaccines[3]	69.1%	76.6%	0.08	1.57*	[1.02,2.43]	1.54*	[1.02,2.32]
All recommended vaccines[4]	36.7%	53.2%	<0.001	1.89**	[1.28,2.81]	1.97***	[1.35,2.89]
Negative immunization outcomes							
Pentavalent zero dose	5.4%	2.3%	0.03	0.37*	[0.16,0.88]	0.38*	[0.18,0.82]
Pentavalent dropout[5]	4.1%	4.6%	0.75	1.02	[0.51,2.01]	1.10	[0.54,2.24]

[1]Reference is children not proximal to a vaccine-providing facility
[2]Models also control for household wealth, mother's education, mother's age, child sex, child birth order, region of country
[3]Defined as BCG 1 dose, OPV 3 doses, DTP/HBV/Hib (pentavalent) 3 doses, measles 1 dose
[4]Basic + OPV birth dose + rotavirus 2 doses + PCV13 3 doses
[5]Dropout denominator is children with at least 1 dose of pentavalent
* p<0.05, ** p<0.01, *** p<0.001

For peer review only

Appendix Table 2a. Logistic regression models of individual-level vaccination among children 12-23 months living in rural areas who are within 5km of a facility providing vaccination. Only facility-characteristic coefficients reported.[1]

	Specific immunization dose coverage											
	BCG		Rotavirus 2 dose		Polio 3 dose		Pentavalent 3 dose		Pneumococcal 3 dose		Measles 1+ dose	
	AOR	95% CI	AOR	95% CI	AOR	95% CI	AOR	95% CI	AOR	95% CI	AOR	95% CI
Facility type offering vaccination [highest available if >1]												
Hospital	*Ref*	*Ref*	*Ref*	*Ref*	*Ref*	*Ref*	*Ref*	*Ref*	*Ref*	*Ref*	*Ref*	*Ref*
Health centre	0.92	[0.34,2.49]	1.03	[0.54,1.95]	1.07	[0.72,1.58]	0.89	[0.48,1.64]	0.69	[0.40,1.20]	1.17	[0.51,2.64]
Health post/clinic/dispensary	1.88	[0.42,8.48]	0.93	[0.38,2.28]	1.94	[0.97,3.86]	0.84	[0.33,2.16]	0.87	[0.34,2.24]	0.79	[0.27,2.34]
Managing authority of facility/ies offering vaccination												
Government												
No	*Ref*	*Ref*	*Ref*	*Ref*	*Ref*	*Ref*	*Ref*	*Ref*	*Ref*	*Ref*	*Ref*	*Ref*
Yes	2.06	[0.51,8.36]	1.65	[0.89,3.07]	2.32**	[1.37,3.93]	1.76	[0.91,3.42]	1.18	[0.64,2.15]	1.34	[0.70,2.55]
CHAM												
No	*Ref*	*Ref*	*Ref*	*Ref*	*Ref*	*Ref*	*Ref*	*Ref*	*Ref*	*Ref*	*Ref*	*Ref*
Yes	1.71	[0.44,6.67]	1.54	[0.80,2.97]	2.47***	[1.46,4.18]	2.04*	[1.06,3.93]	1.05	[0.60,1.86]	1.39	[0.63,3.07]
Private for-profit												
No	*Ref*	*Ref*	*Ref*	*Ref*	*Ref*	*Ref*	*Ref*	*Ref*	*Ref*	*Ref*	*Ref*	*Ref*
Yes	2.85	[0.31,26.02]	9.83*	[1.20,80.47]	0.82	[0.38,1.79]	5.36	[0.95,30.14]	1.96	[0.33,11.49]	1.57	[0.32,7.73]
NGO												
No	*Ref*	*Ref*	*Ref*	*Ref*	*Ref*	*Ref*	*Ref*	*Ref*	*Ref*	*Ref*	*Ref*	*Ref*
Yes	0.53	[0.13,2.18]	1.42	[0.42,4.81]	0.57	[0.26,1.28]	2.14	[0.54,8.44]	0.76	[0.27,2.14]	1.59	[0.46,5.48]

[1]Models also control for household wealth, education, mother's age, child sex, child birth order, region of country
* p<0.05, ** p<0.01, *** p<0.001

For peer review only

Appendix Table 2b. Logistic regression models of individual-level vaccination among children 12-23 months living in rural areas who are within 5km of a facility providing vaccination. Only facility-characteristic coefficients reported.[1]

	Coverage of group of immunizations				*Negative immunization outcomes*			
	All basic vaccines[2]		All recommended vaccines[3]		Pentavalent 0 dose		Pentavalent dropout	
	AOR	95% CI	AOR	95% CI	AOR	95% CI	AOR	95% CI
Facility type offering vaccination [highest available if >1]								
Hospital	*Ref*	*Ref*	*Ref*	*Ref*	*Ref*	*Ref*	*Ref*	*Ref*
Health centre	1.11	[0.75,1.66]	0.79	[0.55,1.14]	1.46	[0.42,5.12]	0.95	[0.49,1.86]
Health post/clinic/dispensary	1.45	[0.75,2.81]	0.93	[0.56,1.54]	0.20	[0.03,1.28]	1.53	[0.57,4.09]
Managing authority of facility/ies offering vaccination								
Government								
No	*Ref*	*Ref*	*Ref*	*Ref*	*Ref*	*Ref*	*Ref*	*Ref*
Yes	1.82*	[1.15,2.88]	1.94**	[1.24,3.03]	0.08***	[0.02,0.33]	0.97	[0.44,2.11]
CHAM								
No	*Ref*	*Ref*	*Ref*	*Ref*	*Ref*	*Ref*	*Ref*	*Ref*
Yes	2.29***	[1.44,3.63]	1.33	[0.86,2.07]	0.11**	[0.02,0.50]	0.64	[0.31,1.32]
Private for-profit								
No	*Ref*	*Ref*	*Ref*	*Ref*	--	--	*Ref*	*Ref*
Yes	0.84	[0.39,1.78]	1.04	[0.48,2.25]	--	--	0.26	[0.04,1.54]
NGO								
No	*Ref*	*Ref*	*Ref*	*Ref*	--	--	*Ref*	*Ref*
Yes	0.60	[0.28,1.28]	0.62	[0.35,1.10]	--	--	0.60	[0.15,2.32]

[1]Models also control for household wealth, education, mother's age, child sex, child birth order, region of country
[2]Defined as BCG 1 dose, OPV 3 dose, DTP/HBV/Hib (pentavalent) 3 dose, measles 1 dose
[3]Basic + OPV birth dose + rotavirus 2 doses + PCV13 3 doses
* p<0.05, ** p<0.01, *** p<0.001

For peer review only

STROBE Statement—Checklist of items that should be included in reports of ***cross-sectional studies***

	Item No	Recommendation	Page No
**Title and abstract**	1	(*a*) Indicate the study's design with a commonly used term in the title or the abstract	1
		(*b*) Provide in the abstract an informative and balanced summary of what was done and what was found	2
**Introduction**			
Background/rationale	2	Explain the scientific background and rationale for the investigation being reported	4-5
Objectives	3	State specific objectives, including any prespecified hypotheses	5
**Methods**			
Study design	4	Present key elements of study design early in the paper	5-6
Setting	5	Describe the setting, locations, and relevant dates, including periods of recruitment, exposure, follow-up, and data collection	5-6
Participants	6	(*a*) Give the eligibility criteria, and the sources and methods of selection of participants	6
Variables	7	Clearly define all outcomes, exposures, predictors, potential confounders, and effect modifiers. Give diagnostic criteria, if applicable	7
Data sources/ measurement	8*	For each variable of interest, give sources of data and details of methods of assessment (measurement). Describe comparability of assessment methods if there is more than one group	7
Bias	9	Describe any efforts to address potential sources of bias	8, 15
Study size	10	Explain how the study size was arrived at	6, 9
Quantitative variables	11	Explain how quantitative variables were handled in the analyses. If applicable, describe which groupings were chosen and why	7
Statistical methods	12	(*a*) Describe all statistical methods, including those used to control for confounding	7-8
		(*b*) Describe any methods used to examine subgroups and interactions	8
		(*c*) Explain how missing data were addressed	7
		(*d*) If applicable, describe analytical methods taking account of sampling strategy	8
		(*e*) Describe any sensitivity analyses	8
**Results**			
Participants	13*	(a) Report numbers of individuals at each stage of study—eg numbers potentially eligible, examined for eligibility, confirmed eligible, included in the study, completing follow-up, and analysed	9
		(b) Give reasons for non-participation at each stage	N/A
		(c) Consider use of a flow diagram	N/A
Descriptive data	14*	(a) Give characteristics of study participants (eg demographic, clinical, social) and information on exposures and potential confounders	9-10
		(b) Indicate number of participants with missing data for each variable of interest	7
Outcome data	15*	Report numbers of outcome events or summary measures	8-9
Main results	16	(*a*) Give unadjusted estimates and, if applicable, confounder-adjusted estimates and their precision (eg, 95% confidence interval). Make clear which confounders were adjusted for and why they were included	10-12

For peer review only

For peer review only - http://bmjopen.bmj.com/site/about/guidelines.xhtml

		(*b*) Report category boundaries when continuous variables were categorized	7
		(*c*) If relevant, consider translating estimates of relative risk into absolute risk for a meaningful time period	N/A
Other analyses	17	Report other analyses done—eg analyses of subgroups and interactions, and sensitivity analyses	13
**Discussion**			
Key results	18	Summarise key results with reference to study objectives	3-14
Limitations	19	Discuss limitations of the study, taking into account sources of potential bias or imprecision. Discuss both direction and magnitude of any potential bias	15
Interpretation	20	Give a cautious overall interpretation of results considering objectives, limitations, multiplicity of analyses, results from similar studies, and other relevant evidence	14-15
Generalisability	21	Discuss the generalisability (external validity) of the study results	14-15
**Other information**			
Funding	22	Give the source of funding and the role of the funders for the present study and, if applicable, for the original study on which the present article is based	13

*Give information separately for exposed and unexposed groups.

Note: An Explanation and Elaboration article discusses each checklist item and gives methodological background and published examples of transparent reporting. The STROBE checklist is best used in conjunction with this article (freely available on the Web sites of PLoS Medicine at http://www.plosmedicine.org/, Annals of Internal Medicine at http://www.annals.org/, and Epidemiology at http://www.epidem.com/). Information on the STROBE Initiative is available at www.strobe-statement.org.

For peer review only

BMJ Open

The association between childhood immunization coverage and proximity to health facilities in rural settings: A cross-sectional analysis of Service Provision Assessment 2013-14 facility data and Demographic and Health Survey 2015-16 individual data in Malawi

Journal:	*BMJ Open*
Manuscript ID	bmjopen-2022-061346.R1
Article Type:	Original research
Date Submitted by the Author:	13-Apr-2022

Complete List of Authors:	Johns, Nicole; World Health Organization, Department of Data and Analytics<br>Hosseinpoor, Ahmad Reza; World Health Organization, Department of Data and Analytics<br>Chisema, Mike; Malawi Ministry of Health<br>Danovaro-Holliday, M Carolina; World Health Organization, Department of Immunization, Vaccines and Biologicals<br>Kirkby, Katherine; World Health Organization, Department of Data and Analytics<br>Schlotheuber, Anne; World Health Organization, Department of Data and Analytics<br>Shibeshi, Messeret; World Health Organization, Inter-Country Support Team for East and Southern Africa<br>Sodha, Samir V.; World Health Organization, Department of Immunization, Vaccines and Biologicals<br>Zimba, Boston; World Health Organization, Malawi Country Office

**Primary Subject Heading**:	Global health
Secondary Subject Heading:	Public health, Paediatrics
Keywords:	PUBLIC HEALTH, Community child health < PAEDIATRICS, Paediatric infectious disease & immunisation < PAEDIATRICS

SCHOLARONE™ Manuscripts

I, the Submitting Author has the right to grant and does grant on behalf of all authors of the Work (as defined in the below author licence), an exclusive licence and/or a non-exclusive licence for contributions from authors who are: i) UK Crown employees; ii) where BMJ has agreed a CC-BY licence shall apply, and/or iii) in accordance with the terms applicable for US Federal Government officers or employees acting as part of their official duties; on a worldwide, perpetual, irrevocable, royalty-free basis to BMJ Publishing Group Ltd ("BMJ") its licensees and where the relevant Journal is co-owned by BMJ to the co-owners of the Journal, to publish the Work in this journal and any other BMJ products and to exploit all rights, as set out in our licence.

The Submitting Author accepts and understands that any supply made under these terms is made by BMJ to the Submitting Author unless you are acting as an employee on behalf of your employer or a postgraduate student of an affiliated institution which is paying any applicable article publishing charge ("APC") for Open Access articles. Where the Submitting Author wishes to make the Work available on an Open Access basis (and intends to pay the relevant APC), the terms of reuse of such Open Access shall be governed by a Creative Commons licence – details of these licences and which Creative Commons licence will apply to this Work are set out in our licence referred to above.

Other than as permitted in any relevant BMJ Author's Self Archiving Policies, I confirm this Work has not been accepted for publication elsewhere, is not being considered for publication elsewhere and does not duplicate material already published. I confirm all authors consent to publication of this Work and authorise the granting of this licence.

For peer review only

The association between childhood immunization coverage and proximity to health facilities in rural settings: A cross-sectional analysis of Service Provision Assessment 2013-14 facility data and Demographic and Health Survey 2015-16 individual data in Malawi

Nicole E. Johns[1], Ahmad Reza Hosseinpoor[1*], Mike Chisema[2], M. Carolina Danovaro-Holliday[3], Katherine Kirkby,[1] Anne Schlotheuber,[1] Messeret Shibeshi,[4] Samir V. Sodha,[3] Boston Zimba[5]

[1]Department of Data and Analytics, World Health Organization, Geneva, Switzerland.
[2]Malawi Ministry of Health, Lilongwe, Malawi.
[3]Department of Immunization, Vaccines and Biologicals, World Health Organization, Geneva, Switzerland.
[4]Inter-Country Support Team for East and Southern Africa, World Health Organization, Harare, Zimbabwe
[5]Malawi Country Office, World Health Organization, Lilongwe, Malawi
*Corresponding author

Corresponding Author:
Ahmad Reza Hosseinpoor
hosseinpoora@who.int
Department of Data and Analytics
Division of Data, Analytics and Delivery for Impact
World Health Organization
20, Avenue Appia, CH-1211 Geneva 27, Switzerland

Manuscript word count: 3987

Key words: Childhood immunization; vaccination; equity; rural; Malawi

For peer review only

ABSTRACT

Objectives: Despite significant progress in childhood vaccination coverage globally, substantial inequality remains. Remote rural populations are recognized as a priority group for immunization service equity. We aimed to link facility and individual data to examine the relationship between distance to services and immunization coverage empirically, specifically using a rural population.

Design & setting: Retrospective cross-sectional analysis of facility data from the 2013-14 Malawi Service Provision Assessment and individual data from the 2015-16 Malawi Demographic and Health Survey, linking children to facilities within a 5-kilometer radius. We examined associations between proximity to health facilities and vaccination receipt via bivariate comparisons and logistic regression models.

Participants: 2740 children aged 12-23 months living in rural areas.

Outcome measures: Immunization coverage for the six vaccines included in the Malawi EPI schedule for children under one at time of study, as well as two composite vaccination indicators (receipt of basic vaccines and receipt of all recommended vaccines), zero-dose pentavalent coverage, and pentavalent dropout.

Findings: 72% (706/977) of facilities offered childhood vaccination services. Among children in rural areas, 61% were proximal to (within 5km) a vaccine-providing facility. Proximity to a vaccine-providing health facility was associated with increased likelihood of having received the rotavirus vaccine (93% vs 88%, p=0.004) and measles vaccine (93% vs 89%, p=0.01) in bivariate tests. In adjusted comparisons, how close a child was to a health facility remained meaningfully associated with how likely they were to have received rotavirus vaccine (AOR 1.63, 95% CI 1.12-2.33) and measles vaccine (AOR 1.62, 95% CI 1.11-2.37).

Conclusion: Proximity to health facilities was significantly associated with likelihood of receipt for some, but not all, vaccines. Our findings reiterate the vulnerability of children residing far from static vaccination services; efforts that specifically target remote rural populations living far from health facilities are warranted to ensure equitable vaccination coverage.

For peer review only

STRENGTHS AND LIMITATIONS OF THE STUDY

- Health facility and individual data were linked to examine immunization service access and equality for rural populations using a nationally-representative dataset.
- Straight-line distance to nearest immunization service was calculated, and the association between proximity to a facility providing services and child-level immunization coverage outcomes were examined.
- Analyses accounted for additional known predictors of immunization coverage such as birth order and household wealth.
- Straight-line distance to nearest services does not reflect actual access, which is tied to road networks, geography, seasonality, vehicle access, and preferred facilities.
- Distance is only one of many factors which render a population hard-to-reach or hard-to-vaccinate.

For peer review only

INTRODUCTION

Despite significant progress in childhood vaccination coverage globally since the establishment of the Expanded Programme on Immunization (EPI) in 1974, substantial inequality in vaccination coverage remains.[1-4] Within countries, immunization coverage tends to be lowest among the most disadvantaged subgroups, including those living in rural areas.[2 5 6] The Equity Reference Group for Immunization has identified remote rural populations (those living furthest from population centers) as one of the most pressing priority areas of work for improving immunization equity.[7 8] The Immunization Agenda 2030 (IA2030) recognizes coverage and equity as strategic priorities and has the stated goal to leave no one behind, i.e. to ensure that hard to reach and marginalized populations are included and centered in immunization policies and initiatives, and to ultimately achieve universal immunization coverage.[9 10] Similarly, Gavi's most recent strategy also centers the theme of leaving no one behind and indicates equity as an organizing principle and key goal.[11] Globally, governments and key stakeholders in immunization are recognizing equity, including reaching remote rural populations, as a central priority.[8]

Immunization services for remote rural populations involve a number of barriers. On the side of the procurement, distribution, and provision of vaccines, barriers include increased marginal costs of administration, challenges of sufficient health worker availability and training, physical access for supply delivery, and cold-chain continuity.[7] One of the largest barriers on the side of the remote rural patient to immunization provision is physical access to static or outreach services. Distance to a health facility is a commonly cited barrier to vaccination in nationally representative studies and systematic reviews.[12-15] Several studies have examined distance empirically, and find significant negative associations between distance to vaccination site and likelihood of vaccination, though all rely on subnational samples.[16-18] One nationally-representative study from Nigeria found that distance to a health facility (whether it provided vaccination services or not) was significantly and negatively associated with receipt of most vaccines.[19] Distance to a health facility that routinely provides immunization services is likely associated with immunization coverage, particularly among rural populations.

The Malawi health system consists of public, private for profit, and private not for profit sectors; the majority of healthcare services are provided by the government, followed by the major religious provider Christian Health Association of Malawi (CHAM).[20] Though vaccination services may be available at all facilities, the majority of children receive vaccinations through government facilities or outreach clinics.[21] In the community, immunization services are most frequently provided by health workers known as health surveillance assistants (HSAs), who provide door-to-door visitations and staff village, outreach, and mobile clinics.[20 22] Routine

immunization services are also supported by other periodic supplementary immunization activities (SIAs).[23-25]

As of the 2015-16 Malawi Demographic and Health Survey (DHS), an estimated 76% (95% CI 74%-78%) of children age 12-23 months had received basic immunizations (defined as one dose of Bacillus Calmette-Guérin (BCG); three doses of oral polio vaccine (OPV); three doses of pentavalent vaccine (diphtheria, tetanus, pertussis [DTP], hepatitis B virus [HBV], and *Haemophilus influenzae* type b [Hib]); and one dose of measles vaccine), a decline from 81% (95% CI 79-83%) in the 2010-11 DHS.[26] Contrary to findings in other sub-Saharan settings and prior research within Malawi, childhood vaccination coverage was in fact higher among children living in rural areas (77%, 95% CI 74%-79%) than children living in urban areas (70%, 95% CI 63%-76%).[12 26] However, such figures conceal inequalities in access to immunization *within* rural populations, where the most remote are still likely under-immunized.[7] Data from the 2015-16 Malawi DHS confirms these within-rural inequalities: coverage of basic vaccines varied by more than 25 percentage points across rural strata, from 65% (95% CI 53%-75%) to 91% (95% CI 84%-95%).[27] Understanding inequities within rural populations is particularly relevant in Malawi, where 84% of the population live in rural areas.[28]

The policy of the Malawi Ministry of Health is that all Malawians should live within 8 kilometers (km) of a public health facility, though as of 2014 more than two million people were estimated to live further than this from care.[29] The 8km policy is intended to ensure "reasonable walking distance" to healthcare, which is particularly relevant given low vehicle ownership (only 4% of households in rural areas had a car, truck, motorcycle or scooter as of the 2015-16 DHS).[27] Several studies from Malawi suggest that residing far from a facility providing vaccination services is a driver of inequitable immunization coverage for remote rural populations.[30 31] Research has documented the relationship between empirically-derived distance to facility and other healthcare services and outcomes in Malawi;[32-37] to our knowledge, no other study has examined empirically-derived distance to facilities providing vaccination services and immunization coverage in Malawi.

In this study, we hypothesize that children living in rural areas aged 12-23 months who are proximal to a vaccine-providing health facility will be significantly more likely to have received immunizations than children who live further than 5km from the nearest vaccine-providing facility. Substantial research has explored individual-level factors associated with vaccination, but less work has demonstrated an association between empirically-derived facility access and immunization coverage, particularly using nationally-representative data. The nature of the data available in Malawi pose a unique opportunity to explore this quantitatively.

METHODS

Data sources

Facility data come from the Malawi 2013-14 DHS Service Provision Assessment (SPA) survey, a census of formal-sector public and private health facilities in Malawi.[38] It includes all static sites but not outreach clinic locations. Facilities included in the survey were hospitals, health centres, dispensaries, clinics, and health posts; managing authorities included government, CHAM and other faith-based organizations, private sector for-profit organizations, non-governmental organizations (NGOs), and companies.

We used data from the Malawi 2015-16 DHS, the most recent available DHS survey in the country and the closest in time to the SPA, to determine immunization coverage among children aged 12-23 months residing in rural areas. Vaccine doses included were those six in the Malawi EPI schedule for children under one at the time of data collection: BCG, OPV, pentavalent, pneumococcal conjugate vaccine 13-valent (PCV13), rotavirus vaccine, and measles-containing vaccine (Table 1).[39] Details regarding DHS sampling, implementation, and content are published elsewhere.[26] Individual, family, and community characteristics were also extracted from this dataset.

Table 1. Malawi EPI vaccination schedule for children under one year, 2015[1]

Age	Vaccine
Birth up to two weeks after birth	BCG<br>Birth dose OPV
6 weeks	OPV dose 1<br>Pentavalent dose 1<br>PCV13 dose 1<br>Rotavirus dose 1
10 weeks	OPV dose 2<br>Pentavalent dose 2<br>PCV13 dose 2<br>Rotavirus dose 2
14 weeks	OPV dose 3<br>Pentavalent dose 3<br>PCV13 dose 3
9 months	Measles dose 1

[1]Though this schedule reflects the EPI schedule at time of data analyzed here, several changes to the EPI have gone into effect since 2015. The measles vaccine was replaced with measles and rubella vaccine in July 2017. The trivalent OPV was replaced with bivalent OPV in 2016, and inactivated polio vaccine (IPV) was introduced starting in December 2018. The malaria vaccine (RTS,S/ASO1) was also piloted in 2019 but is not yet in the national EPI vaccination schedule as of April 2022.

For peer review only - http://bmjopen.bmj.com/site/about/guidelines.xhtml

Geographic data linkage

Facility geo-location, provided as part of SPA data, was matched to individual data using the DHS-suggested technique of a Euclidian (straight-line) distance buffer around each DHS cluster centroid.[40] This buffer is defined as 5km in rural areas; this corresponds with the DHS offset of cluster GPS locations by up to 5km in rural areas. Therefore, we defined proximity to a vaccine-providing facility as children living in rural areas within 5km of a facility that reported regularly providing vaccination services on-site and/or via outreach. We also tested alternate buffer distances, including 8km based on Malawi Ministry of Health definitions (see sensitivity analysis description below).[29]

Measures

Facility data indicated vaccination provision, stock, fees, and preparedness. For the sake of these analyses, only presence of a health facility offering vaccination services was used. Indicators of vaccination fees were not used because very few facilities (2%) reported charging vaccination-specific fees. Indicators of full EPI provision were not used because most facilities offering any vaccination services offered all EPI vaccines (99% of facilities offering vaccines offered full EPI). Despite a 2-3 year gap between the survey of facilities (2013-14) and survey of individuals (2015-16, children born October 2013-February 2015), retroactive reporting of immunization for children 12-23 months reflects immunization receipt close to the time of facility survey, and provision of any vaccination services is likely to have remained constant over that time.

Immunization coverage was defined as the proportion of children aged 12–23 months who received the indicated vaccine dose by the time of survey. For OPV, pentavalent, and PCV13 vaccines, children were considered having received the complete series of each vaccine type if they had three doses. Birth dose OPV was considered separately from the three-dose OPV series. For rotavirus vaccine, two doses were considered the complete series, and for BCG and measles, one dose (or more) was considered complete. We also examined receipt of basic vaccines, also termed full immunization with basic vaccine doses, (one dose BCG, three doses OPV, three doses pentavalent, and one dose measles) as well as receipt of all recommended vaccines (basic vaccine doses plus birth dose OPV, two doses rotavirus, and three doses PCV13). Finally, we created indicators of zero-dose pentavalent coverage (no receipt of pentavalent vaccine) and pentavalent dropout (receipt of the first dose, but not the third dose, of pentavalent vaccine). For all vaccines, both mother's recall and vaccination card verification (with or without date) were used to determine coverage. Similarly, vaccines received within the recommended time frame and any time after were included. The few 'Don't know' responses

were coded as non-receipt of the corresponding vaccine/dose (<1% for all, N=0 to 10 per vaccine dose).

Analyses

All analyses were limited to rural populations, as defined by DHS.[41] We first used bivariate Pearson's chi-squared tests to assess associations between vaccination coverage and proximity to a vaccine-providing health facility among children living in rural areas, for all immunization outcomes. We then constructed unadjusted and adjusted logistic regression models to assess the association between proximity to a vaccine-providing health facility and immunization outcomes among children living in rural areas, with adjusted models controlling for: child's sex [male, female], birth order [1, 2, 3, 4, 5+], household wealth [quintile], mother's age [years], mother's education [none, primary, secondary or higher], and subnational region [Northern, Central, Southern]. All covariates were selected *a priori* based on established association with likelihood of immunization and availability in DHS survey data.[21 31 42]

We then conducted several sensitivity analyses. We constructed multinomial models to separate immunization verified by vaccine card and immunization noted via mother's recall, to examine whether the association between facility proximity and immunization was sensitive to the nature of vaccination ascertainment. For each vaccine, we defined a 3-level outcome as: not received (referent group), received and verified by vaccine card, and received as noted via mother's recall. We also modified to the proximity radius to 8km rather than the 5km suggested by DHS, to align with Malawi Ministry of Health policy and assess whether findings were sensitive to the distance radius used.[29 40] Finally, we restricted the sample to children residing within 5km of a vaccine-providing facility, and examined whether facility level of care and managing authority were associated with vaccination coverage in adjusted logistic regression models. We captured facility level of care via a single variable, classified as: hospital, health centre, or health post/clinic/dispensary. We assigned children who were within 5km of multiple facilities providing vaccines to the highest level of care among those proximal facilities. We captured facility managing authority via a set of four indicators, separately assessing whether a child was proximal to a vaccine-providing facility managed by the government, CHAM, private for-profit, and/or NGO.

All individual-level analyses accounted for complex sampling design using provided DHS survey weights. Statistical significance was set at $p<0.05$ for Pearson's chi-squared tests, Adjusted Odds Ratios (AORs), and adjusted Relative Risk Ratios (aRRRs); 95% confidence intervals (CIs) are reported throughout. We conducted analyses using ArcGIS Pro 2.8.0 and STATA 15.1.

Ethics Approval

For peer review only - http://bmjopen.bmj.com/site/about/guidelines.xhtml

Ethical approval for data collection was obtained by the DHS Program and implementing partners at time of survey, and participants provided informed consent to participate in the DHS survey at the time of data collection. This secondary data analysis using publicly-available de-identified data was reviewed and approved by the National Health Sciences Research Committee of Malawi (#20220106).

Patient and public involvement

Due to the nature of this analysis, patients and the public were not involved in the design, conduct, reporting, or dissemination of this research.

RESULTS

Of 977 facilities surveyed, 72% (N=706) offered any childhood immunization services (Table 2).

Table 2. Health facilities offering childhood immunization services, Malawi SPA 2013-14.

	N	%
Total	*706*	*100.0%*
Type of vaccination offerings		
% offering all EPI vaccines	696	98.6%
Facility type		
Central hospital	1	0.1%
District hospital	24	3.4%
Rural/community hospital	41	5.8%
Other hospital	31	4.4%
Health centre	465	65.9%
Maternity	3	0.4%
Dispensary	40	5.7%
Clinic	81	11.5%
Health post	20	2.8%
Managing authority		
Government/public	457	64.7%
Christian Health Association of Malawi (CHAM)	153	21.7%
Private for profit	39	5.5%
Other mission/faith-based	6	0.9%
Non-governmental organization (NGO)	19	2.7%
Company	32	4.5%

Individual survey data included 2740 children aged 12-23 months living in rural areas (Appendix Table 1). All living children aged 12-23 months residing in rural areas were included. The majority of children were proximal to health facilities: 64% were within 5km of *any* health

facility and 61% were within 5km of a vaccine-providing facility (Table 3). Proximity was often limited to a single facility; only 5% were within 5km of two or more vaccine-providing facilities.

Overall, immunization coverage among children in rural areas was above 80% for all examined individual vaccines: 97.5% 1-dose BCG, 82.2% 3-dose OPV, 93.4% 3-dose pentavalent, 91.1% 2-dose rotavirus, 89.7% 3-dose PCV13, and 91.7% 1-dose measles (Table 3). However, full immunization coverage was lower: three-fourths (76.8%) of children had basic vaccine doses and only half (50.4%) had all vaccines recommended for children under one. Few children received no doses of pentavalent vaccine (2.5%), and among children who started their pentavalent vaccine series, 4.2% did not complete it (indicating dropout). Among children with vaccination date information available, all vaccines except measles had 99% or greater receipt within the first year of life, while 93.4% of measles first doses were received before age one (results not shown).

Table 3. Study sample - children aged 12-23 months in rural areas, Malawi DHS 2015-16.

	Unweighted N	Weighted %
Total	*2740*	*100.0%*
**Facility proximity indicators**		
Facility proximity within 5km		
No facility within 5km	978	36.3%
Any facility within 5km	*1762*	*63.7%*
Vaccine-providing facility within 5km	1698	61.2%
Non-vaccine-providing facility only within 5km	64	2.6%
Proximal to vaccine-providing facility, by type[1]		
Hospital	403	12.1%
Health centre	1264	47.2%
Health post/clinic/dispensary	289	10.9%
Proximal to vaccine-providing facility, by managing authority[1]		
Government/public	1305	46.7%
CHAM	501	16.9%
Private for profit	53	2.8%
NGO	74	2.8%
**Immunization coverage indicators**		
Specific immunization dose coverage		
BCG	2673	97.5%
OPV 3 doses	2273	82.2%
Pentavalent 3 doses	2559	93.4%
Rotavirus 2 doses	2510	91.1%
PCV13 3 doses	2460	89.7%
Measles 1+ dose	2524	91.7%

For peer review only - http://bmjopen.bmj.com/site/about/guidelines.xhtml

Coverage of group of immunizations		
Basic vaccines[2]	2129	76.8%
All recommended vaccines[3]	1450	50.4%
Negative immunization outcomes		
Pentavalent zero dose	61	2.5%
Pentavalent dropout[4]	120	4.2%

[1]Children could be proximal to more than one vaccine-providing facility and thus more than one vaccine-providing facility type/managing authority

[2]Defined as BCG 1 dose, OPV 3 doses, DTP/HBV/Hib (pentavalent) 3 doses, measles 1 dose

[3]Basic + OPV birth dose + rotavirus 2 doses + PCV13 3 doses

[4]Dropout denominator is children with at least 1 dose of pentavalent; represents percent of children receiving first but not third dose of pentavalent.

Children living in rural areas who were proximal to a vaccine-providing facility were more likely to have some, but not all, vaccine doses in unadjusted bivariate tests when compared to children who were not in proximity to a vaccine-providing facility: rotavirus (93% vs 88%, p=0.004), measles (93% vs 89%, p=0.01), and full immunization with basic vaccines (79% vs 74%, p=0.04) and with all recommended vaccines (54% vs 44%, p<0.001) (Table 4). No significant bivariate differences were observed for BCG, OPV, pentavalent, or PCV13 vaccine, nor pentavalent zero dose or dropout.

These relationships hold true in fully adjusted regression models (Table 4). Compared to children living greater than 5km from the nearest facility providing immunization services, children living proximal to a vaccine-providing facility had greater odds of receiving rotavirus (AOR 1.63, 95% CI 1.12-2.33) and measles (AOR 1.62, 95% CI 1.11-2.37) vaccines. They also had marginally greater odds of receiving basic vaccines (AOR 1.28, 95% CI 0.99-1.65, p=0.057) and greater odds of receiving all recommended vaccines (AOR 1.38, 95% CI 1.09-1.74). They had somewhat lower odds of being zero-dose for pentavalent (AOR 0.53, 95% CI 0.28-1.01, p=0.052). No statistically significant associations were observed with BCG, OPV, pentavalent, or PCV13 receipt, nor pentavalent dropout.

Table 4. Unadjusted and adjusted comparisons of immunization rates among children in rural areas aged 12-23 months by presence of vaccine-providing facility in 5km radius, Malawi 2013-14.

	Unadjusted rates			Unadjusted logistic regression		Adjusted logistic regression		Adjusted multinomial logistic regression, three-level recording-method-specific outcome			
								Mother recall		Vaccine card	
	No vaccine-providing facility proximal	Vaccine-providing facility proximal	p-value	OR[1]	95% CI	AOR[1,2]	95% CI	aRRR[1,2]	95% CI	aRRR[1,2]	95% CI
Specific immunization dose coverage											
BCG	97.4%	97.6%	0.78	1.09	[0.58,2.05]	1.17	[0.63,2.20]	1.28	[0.67,2.42]	1.15	[0.61,2.16]
Rotavirus 2 doses	88.5%	92.8%	0.004	1.68**	[1.17,2.41]	1.63**	[1.13,2.33]	1.71*	[1.12,2.60]	1.72**	[1.18,2.51]
OPV 3 doses	81.4%	82.6%	0.55	1.09	[0.83,1.42]	1.08	[0.83,1.40]	1.15	[0.75,1.75]	1.11	[0.85,1.46]
Pentavalent 3 doses	92.6%	94.0%	0.30	1.24	[0.83,1.87]	1.23	[0.84,1.82]	1.33	[0.85,2.09]	1.20	[0.80,1.79]
PCV13 3 doses	88.9%	90.2%	0.49	1.15	[0.78,1.68]	1.07	[0.75,1.53]	1.09	[0.70,1.68]	1.1	[0.76,1.59]
Measles 1+ dose	89.3%	93.3%	0.01	1.68**	[1.14,2.48]	1.62*	[1.11,2.37]	1.52*	[1.04,2.24]	1.59*	[1.07,2.35]
Coverage of group of immunizations											
Basic vaccines[3]	73.8%	78.7%	0.04	1.31*	[1.01,1.69]	1.28	[0.99,1.65]	--	--	--	--
All recommended vaccines[4]	45.0%	53.7%	<0.001	1.42**	[1.13,1.78]	1.38**	[1.09,1.74]	--	--	--	--
Negative immunization outcomes											
Pentavalent zero dose	3.4%	1.9%	0.08	0.55	[0.28,1.10]	0.53	[0.28,1.01]	--	--	--	--
Pentavalent dropout[5]	4.1%	4.2%	0.91	1.02	[0.67,1.56]	1.05	[0.69,1.61]	--	--	--	--

[1]Reference is children not proximal to a vaccine-providing facility
[2]Models also control for household wealth, mother's education, mother's age, child sex, child birth order, region of country
[3]Defined as BCG 1 dose, OPV 3 doses, DTP/HBV/Hib (pentavalent) 3 doses, measles 1 dose
[4]Basic + OPV birth dose + rotavirus 2 dose + PCV13 3 dose
[5]Dropout denominator is children with at least 1 dose of pentavalent
* p<0.05, ** p<0.01, *** p<0.001

For peer review only

Associations between proximity to a vaccine-providing facility and immunization coverage were similar for both mother's recall and vaccination card documented immunization in multinomial models (Table 4). The majority of children in rural areas (80%, 95% CI 78%-82%) had a vaccination card which was seen. Children were both more likely to have vaccination card-recorded rotavirus vaccination (AOR 1.71, 95% CI 1.12-2.60) or mother-recalled rotavirus vaccination (AOR 1.72, 95% CI 1.18-2.51) if they were proximal to a vaccine-providing facility. Children were also both more likely to have vaccination card recorded measles vaccination (AOR 1.59, 95% CI 1.07-2.35) or mother-recalled measles vaccination (AOR 1.52, 95% CI 1.04-2.24) if they were proximal to a vaccine-providing facility. No significant associations between proximity to vaccine-providing facility and immunization, whether ascertained through mother recall or vaccination card, were observed for BCG, OPV, pentavalent, or PCV13 vaccines.

When considering proximity as 8km rather than 5km, 87% of children were proximal to a vaccine-providing facility; associations with immunization coverage were similar to those with 5km proximity definition (Appendix Table 2). We observed significantly greater immunization coverage among children within 8km of a vaccine-providing facility for rotavirus (91.9% vs 87.0%, p=0.02), measles (92.0% vs 86.0%, p=0.03), and all recommended vaccines (53.2% vs 36.7%, p=0.001) in unadjusted comparisons. Additionally, zero-dose pentavalent receipt was significantly less common among children within 8km of a vaccine-providing facility compared to those not (2.3% vs 5.4%, p=0.03). Findings were similar in adjusted models: measles (AOR 1.88, 95% CI 1.06-3.33, p=0.03), basic vaccines (AOR 1.54, 95% CI 1.02-2.32, p=0.04), all recommended vaccines (AOR 1.97, 95% CI 1.35-2.89, p<0.001), zero-dose pentavalent (AOR 0.38, 95% CI 0.18-0.83, p=0.01). Rotavirus vaccine did not have a significant association with 8km proximity to a vaccine-providing facility in adjusted models (AOR 1.53, 95% CI 0.95-2.45, p=0.08). As with 5km findings, no significant associations were observed for OPV, BCG, pentavalent, or PCV13 vaccines.

When limited to children within 5km of a vaccine-providing facility, the level of the facility was not associated with any of the immunization indicators (Appendix Tables 3a, 3b). Proximity to a government-run facility offering vaccination services was associated with greater odds of receipt of 3-dose OPV (AOR 2.32, 95% CI 1.37-3.93), basic vaccines (AOR 1.82, 95% CI 1.15-2.88), and all recommended vaccines (AOR 1.94, 95% CI 1.24-3.03), and lower odds of zero-dose pentavalent (AOR 0.08, 95% CI 0.02-0.33). Proximity to a CHAM facility offering vaccination services was associated with greater odds of receipt of 3-dose OPV (AOR 2.47, 95% CI 1.46-4.18), 3-dose pentavalent (AOR 2.04, 95% CI 1.05-3.93), and basic vaccines (AOR 2.29, 95% CI 1.44-3.63), and lower odds of zero-dose pentavalent (AOR 0.11, 95% CI 0.02-0.50).

DISCUSSION

Among children aged 12-23 months living in rural areas, proximity to a health facility providing vaccination services was associated with increased likelihood of rotavirus and measles vaccine receipt (and therefore receipt of all recommended vaccines, as rotavirus and measles are part of this composite indicator), as well as with decreased likelihood of zero-dose pentavalent vaccination. Even when accounting for known child, mother, family, and geographic determinants of immunization, how close a child was to a health facility was meaningfully associated with how likely they were to have received certain vaccines.

Proximity to a vaccine-providing health facility among rural children can be considered a proxy for remote ruralness: those who are far from a vaccine-providing facility are also likely to be far from a population center

For peer review only - http://bmjopen.bmj.com/site/about/guidelines.xhtml

and far from services more generally. Therefore, these findings indicate that remote rural children in Malawi were likely inequitably under-vaccinated at the time of the 2015-16 DHS. This is an important equity consideration; despite higher immunization coverage amongst rural children than urban children overall, these findings reiterate that rural populations are not a monolith and that inequities are present beyond urban/rural differences. While geographic distance is just one of many factors which render a population hard-to-reach or hard-to-vaccinate, it is relatively easy to define and target, and is a meaningful correlate of coverage.[43] Equity-focused interventions and monitoring efforts should therefore use as granular geographic delineations as possible, with particular focus on those populations furthest from care.

We observed differential associations by type of immunization. The measles vaccine is delivered on its own several months after other infant vaccinations, and the requirement of a specific healthcare visit to obtain it may exacerbate the barrier of increased distance for accessing care. While rotavirus vaccine is offered simultaneously with other vaccinations, it was introduced to the Malawi EPI in October 2012 and outreach efforts may still have been in scale-up at the time the children under consideration were eligible for vaccination. It was introduced with strict age restrictions (first dose at 6-12 weeks, 4 weeks between doses, second dose no later than 16 weeks), which were not formally removed from the Malawi EPI until 2017.[44] The narrow time range for vaccination created by these limits may have made the vaccine harder to access for populations far from facilities. Conversely, the null findings for 3-dose OPV and 3-dose pentavalent vaccine add evidence to the success of the Malawi EPI in ensuring access to these vaccines among rural populations more broadly.[39]

In analyses restricted to children living within 5km of vaccine-providing facilities, we observed no differences in immunization coverage by facility level of care, but we did find significant differences by facility managing authority. Similar odds of immunization regardless of facility level are unsurprising given that immunization services do not require highly specialized equipment or intensive provider training. Greater likelihood of immunization when the proximal facility was managed by the government or CHAM likely reflects the higher rate of outreach efforts performed by these authorities, the lack of fees for immunization at these facilities, and greater resource availability, training, and oversight more broadly.[45] Additional public-sector outreach efforts in areas where the only health facilities are run privately may thus be warranted.

Our finding that immunization coverage is inequitably lower among children further from health facilities is particularly relevant in light of the COVID-19 pandemic and its effects on childhood immunization services. Pandemic effects on childhood immunization services include service provision limitations such as suspension of outreach activities, disruption and suspension of in-facility services, disruption to vaccine and supply availability, shortages of available healthcare workers, and service utilization limitations including travel restrictions, concern for health and safety in seeking services, and lack of knowledge of service availability.[46 47] Mitigation efforts within Malawi and in other country contexts reduced the disruptions to routine immunization, resulting in only a 1-2% decline in coverage of vaccinations at the national level for 2020.[48-50] However, remote rural patients are most likely to have experienced these disruptions, given the reliance on outreach services or on travel to seek care. Strategies will be needed to ensure that missed children are caught-up for equitable immunization coverage.[51]

Limitations

These findings must be considered in light of several limitations. First, individual data does not indicate where vaccination services were rendered; children may have received immunization services from an alternate facility than the one most proximal to them (including via outreach, the locations of which were not assessed in the SPA). Thus, it would be inappropriate to suggest a causal relationship between the most proximal facility's immunization service availability and an individual child's immunization. Additionally, outreach services are widely used in this population, with more than 5000 fixed and mobile outreach clinics throughout the country;[39] the observed association of immunization coverage with distance to static clinics likely underestimates the true strength of association with distance to location where vaccination was actually received. Second, general service availability may not reflect actual service readiness and availability at the time of immunization receipt. Third, three planned sensitivity analyses were not possible: separately analyzing facilities offering within-facility and outreach services was not possible as 99% of children who were proximal to a vaccine-providing facility were proximal to one offering both in-facility and outreach services; examining specific vaccination availability was not possible because 99% of children who were proximal to a vaccine-providing facility were proximal to one offering all six examined vaccines; examining receipt of vaccines within vs after the first year was not possible because >99% of children who had vaccination dates available received all vaccines by age one (with the exception of measles vaccine, which had 93% receipt by age one). Fourth, while the most recent available surveys were used, at time of publication these data are now 6-9 years old; additional research using more recent data will add insight into current realities. Finally, the use of buffer distances does not account for actual travel distance, geography, or time, nor does it account for seasonal differences in physical access (e.g. due to rains). Similarly, the use of DHS cluster centroids does not reflect the actual household geolocation. Statistically, this will bias findings toward the null as the buffer distance used will not be precise; any associations observed are likely underestimates of the true strength of association. Recent studies support the use of other methods such as theoretical catchment areas for more accurate facility linkage.[52] However, given the research question and the variable size of administrative and catchment areas, buffer distance was considered appropriate for these analyses.

CONCLUSION

Findings from this study align with previous work demonstrating a significant association between immunization coverage and distance to vaccine-providing facilities,[16-19] and expand on this by utilizing a nationally-representative sample with a focus on children living in rural areas. Remote rural populations have been identified as a key target for improving immunization equity, and these findings reiterate the vulnerability of children residing far from static vaccination services. Efforts that target remote rural populations living far from health facilities, even using crude measures of identification such as straight-line distance from facilities, are warranted to ensure equitable vaccination coverage. These analyses also suggest that health facility-level data can and should be used for further analyses of inequalities in immunization.

For peer review only

Abbreviations

AOR: adjusted odds ratio; aRRR: adjusted relative risk ratio; BCG: *Bacillus Calmette-Guérin*; CHAM: Christian Health Association of Malawi; CI: confidence interval; DHS: Demographic and Health Survey; DTP: diphtheria, tetanus, pertussis vaccine; EPI: Expanded Programme on Immunization; GPS: global positioning system; HBV: hepatitis B virus; Hib: *Haemophilus influenzae* type b; KM: kilometer; NGO: non-governmental organization; OPV: oral polio vaccine; PCV13: pneumococcal conjugate vaccine 13-valent; SIA: supplementary immunization activity; SPA: Service Provision Assessment

Funding

Funding for this work was provided by Gavi, the Vaccine Alliance. The funding source had no role in the study design, analysis, interpretation of data, or decision to publish results.

Disclaimer

The authors alone are responsible for the views expressed in this article and they do not necessarily represent the views, decisions or policies of the institutions with which they are affiliated.

Data availability

Data for this study are publicly available upon request through the DHS Program. The data are owned by the Government of Malawi and are archived and managed by The DHS Program. Interested researchers must register at https://dhsprogram.com/data/ and can then request permission to download the SPA and DHS datasets.

Analytical code for results presented here is available upon reasonable request from the corresponding author.

Conflicts of interests

The authors have no conflicts of interest to disclose.

Contributors

AH led initial study conceptualization and provided oversight of analyses and writing. NJ generated specific hypotheses, led data analyses, and created initial manuscript draft. MC, MS, and BZ provided country-specific context, content, and feedback for interpretation of the data. CD-H and SS provided immunization context, content, and feedback for interpretation of the data. KK and AS provided health equity context, content, and feedback for interpretation of the data. All authors contributed to substantive review and revision of the final manuscript, provided final approval of the version to be published, and agree to be accountable for all aspects of the work.

For peer review only

REFERENCES

1. Keja K, Chan C, Hayden G, et al. Expanded programme on immunization. *World health statistics quarterly Rapport trimestriel de statistiques sanitaires mondiales* 1988;41(2):59-63.
2. State of inequality: childhood immunization. Geneva: World Health Organization, 2016.
3. Galles NC, Liu PY, Updike RL, et al. Measuring routine childhood vaccination coverage in 204 countries and territories, 1980–2019: a systematic analysis for the Global Burden of Disease Study 2020, Release 1. *The Lancet* 2021;398(10299):503-21.
4. Muhoza P, Danovaro-Holliday MC, Diallo MS, et al. Routine Vaccination Coverage - Worldwide, 2020. *Mmwr-Morbid Mortal W* 2021;70(43):1495-500.
5. Explorations of inequality: Childhood immunization. Geneva, Switzerland: World Health Organization, 2018.
6. Hosseinpoor AR, Bergen N, Schlotheuber A, et al. State of inequality in diphtheria-tetanus-pertussis immunisation coverage in low-income and middle-income countries: a multicountry study of household health surveys. *The Lancet Global Health* 2016;4(9):e617-e26.
7. Levine O, Lemango E, Befson J, et al. ERG Discussion Paper 08: Tackling inequities in immunization outcomes in remote rural contexts. 2018. https://sites.google.com/view/erg4immunisation/discussion-papers (accessed 09/21/21).
8. Chopra M, Bhutta Z, Blanc DC, et al. Addressing the persistent inequities in immunization coverage. *B World Health Organ* 2020;98(2):146-48. doi: 10.2471/Blt.19.241620
9. World Health Organization. Immunization Agenda 2030: a global strategy to leave no one behind. *Geneva: WHO* 2020
10. Lindstrand A, Cherian T, Chang-Blanc D, et al. The World of Immunization: Achievements, Challenges, and Strategic Vision for the Next Decade. *The Journal of infectious diseases* 2021;224(Supplement_4):S452-S67.
11. Gavi The Vaccine Alliance. Phase V (2021–2025) 2019 [updated 9 Jun 2021. Available from: https://www.gavi.org/our-alliance/strategy/phase-5-2021-2025.
12. Bangura JB, Xiao S, Qiu D, et al. Barriers to childhood immunization in sub-Saharan Africa: A systematic review. *BMC Public Health* 2020;20(1):1108. doi: 10.1186/s12889-020-09169-4 [published Online First: 2020/07/16]
13. Rahman M, Obaida-Nasrin S. Factors affecting acceptance of complete immunization coverage of children under five years in rural Bangladesh. *Salud Publica Mex* 2010;52(2):134-40. doi: 10.1590/s0036-36342010000200005
14. Adedokun ST, Uthman OA, Adekanmbi VT, et al. Incomplete childhood immunization in Nigeria: a multilevel analysis of individual and contextual factors. *BMC Public Health* 2017;17(1):236. doi: 10.1186/s12889-017-4137-7
15. Phillips DE, Dieleman JL, Lim SS, et al. Determinants of effective vaccine coverage in low and middle-income countries: a systematic review and interpretive synthesis. *Bmc Health Serv Res* 2017;17 doi: ARTN 681
16. Tefera YA, Wagner AL, Mekonen EB, et al. Predictors and Barriers to Full Vaccination among Children in Ethiopia. *Vaccines* 2018;6(2) doi: 10.3390/vaccines6020022
17. Miyahara R, Jasseh M, Gomez P, et al. Barriers to timely administration of birth dose vaccines in The Gambia, West Africa. *Vaccine* 2016;34(29):3335-41. doi: 10.1016/j.vaccine.2016.05.017 [published Online First: 2016/05/20]
18. Kiptoo E, Esilaba M, Kobia G, et al. Factors influencing low immunization coverage among children between 12-23 months in East Pokot, Baringo Country, Kenya. *Int J Vaccines* 2015;1(2):00012.
19. Sato R. Association between access to a health facility and continuum of vaccination behaviors among Nigerian children. *Hum Vacc Immunother* 2020;16(5):1215-20. doi: 10.1080/21645515.2019.1678360
20. Ministry of Health. Financial Sustainability Plan (FSP) for Expanded Programme on Immunization. Linongwe, Malawi, 2004.
21. Munthali AC. Determinants of vaccination coverage in Malawi: evidence from the demographic and health surveys. *Malawi Med J* 2007;19(2):79-82. doi: 10.4314/mmj.v19i2.10934 [published Online First: 2007/06/01]
22. Nyirenda L, Flikke R. Frontline Vaccinators and Immunisation Coverage in Malawi. *Forum Dev Stud* 2013;40(1):27-46. doi: 10.1080/08039410.2012.725676
23. Minetti A, Kagoli M, Katsulukuta A, et al. Lessons and challenges for measles control from unexpected large outbreak, Malawi. *Emerg Infect Dis* 2013;19(2):202-9. doi: 10.3201/eid1902.120301
24. Chinele J. Over a million children get protected against polio in Malawi. 2021 26 August 2021. https://www.gavi.org/vaccineswork/over-million-children-get-protected-against-polio-malawi (accessed 09/21/2021).

For peer review only

25. Kainga HW, Ssendagire S, Ssanyu JN, et al. Proportion of children aged 9-59 months reached by the 2017 measles supplementary immunization activity among the children with or without history of measles vaccination in Lilongwe district, Malawi. *PLoS One* 2021;16(1):e0243137. doi: 10.1371/journal.pone.0243137
26. National Statistical Office (NSO) [Malawi] and ICF. Malawi Demographic and Health Survey 2015-16. Zomba, Malawi, and Rockville, Maryland, USA.: NSO and ICF., 2017.
27. National Statistical Office (NSO) [Malawi] and ICF. Malawi Demographic and Health Survey 2015-16 [Dataset]. In: National Statistical Office (NSO) [Malawi] and ICF [Producers], ed.: ICF [Distributor], 2017.
28. 2018 Population and Housing Census Main Report. Zomba, Malawi: Malawi National Statistical Office, 2019.
29. Ministry of Health. 2014/15 ANNUAL REVIEW REPORT FOR THE HEALTH SECTOR. Linongwe, Malawi, 2015.
30. Okeibunor JC, Ogbuanu I, Blanche A, et al. Towards a Strategy for Reducing Missed Opportunities for Vaccination in Malawi: Implications of a Qualitative Health Facility Assessment. *J Immunol Sci* 2018;Suppl(7):46-54.
31. Ntenda PAM, Nkoka O, Nana AW, et al. Factors associated with completion of childhood immunization in Malawi: a multilevel analysis of the 2015-16 Malawi demographic and health survey. *Trans R Soc Trop Med Hyg* 2019 doi: 10.1093/trstmh/trz029
32. McGuire F, Kreif N, Smith PC. The effect of distance on maternal institutional delivery choice: Evidence from Malawi. *Health Econ* 2021;30(9):2144-67. doi: 10.1002/hec.4368
33. Leslie HH, Fink G, Nsona H, et al. Obstetric Facility Quality and Newborn Mortality in Malawi: A Cross-Sectional Study. *PLoS Med* 2016;13(10):e1002151. doi: 10.1371/journal.pmed.1002151
34. Peven K, Taylor C, Purssell E, et al. Distance to available services for newborns at facilities in Malawi: A secondary analysis of survey and health facility data. *PloS one* 2021;16(7):e0254083.
35. Mallick L, Benedict RK, Wang W. Facility readiness and counseling during antenatal care and the relationship with early breastfeeding in Haiti and Malawi. *BMC Pregnancy Childbirth* 2020;20(1):325. doi: 10.1186/s12884-020-02919-7
36. Benedict R, Wang W, Mallick L. Examining the Role of Health Facilities in Supporting Iron Folic Acid Supplementation Adherence Among Women in Malawi (OR25-03-19). *Current developments in nutrition* 2019;3(Supplement_1):nzz051. OR25-03-19.
37. Digitale J, Psaki S, Soler-Hampejsek E, et al. Correlates of contraceptive use and health facility choice among young women in Malawi. *The ANNALS of the American Academy of Political and Social Science* 2017;669(1):93-124.
38. Ministry of Health - MoH/Malawi and ICF International. Malawi Service Provision Asessment (MSPA) 2013-14. Lilongwe, Malawi: MOH/Malawi and ICF International, 2014.
39. EPI Comprehensive Multi-Year Plan 2016-2020. 2015. https://extranet.who.int/countryplanningcycles/sites/default/files/planning_cycle_repository/malawi/malawi_cmyp_2016-2020.pdf (accessed 09/21/2021).
40. Burgert CR, Prosnitz D. Linking DHS Household and SPA Facility Surveys: Data Considerations and Geospatial Methods. DHS Spatial Analysis Reports No 10. Rockville, Maryland, USA: ICF, 2014.
41. Fish TD, Janocha B, Dontamsetti T, et al. GEOSPATIAL COVARIATES: PROXIES FOR MAPPING URBAN-RELATED INDICATORS. DHS SPATIAL ANALYSIS REPORTS 19. Rockville, Maryland, USA: ICF, 2020.
42. Wiysonge CS, Uthman OA, Ndumbe PM, et al. Individual and contextual factors associated with low childhood immunisation coverage in sub-Saharan Africa: a multilevel analysis. *PloS one* 2012;7(5):e37905.
43. Ozawa S, Yemeke TT, Evans DR, et al. Defining hard-to-reach populations for vaccination. *Vaccine* 2019;37(37):5525-34. doi: 10.1016/j.vaccine.2019.06.081
44. Mandomando I, Mumba M, Biey JNM, et al. Implementation of the World Health Organization recommendation on the use of rotavirus vaccine without age restriction by African countries. *Vaccine* 2021;39(23):3111-19. doi: 10.1016/j.vaccine.2021.03.021
45. Makwero MT. Delivery of primary health care in Malawi. *Afr J Prim Health Ca* 2018;10(1) doi: ARTN a1799
46. Olorunsaiye CZ, Yusuf KK, Reinhart K, et al. COVID-19 and Child Vaccination: A Systematic Approach to Closing the Immunization Gap. *International Journal of Maternal and Child Health and AIDS* 2020;9(3):381.
47. Shet A, Carr K, Danovaro-Holliday MC, et al. Impact of the SARS-CoV-2 pandemic on routine immunisation services: evidence of disruption and recovery from 170 countries and territories. *Lancet Glob Health* 2021 doi: 10.1016/S2214-109X(21)00512-X
48. Dixit SM, Sarr M, Gueye DM, et al. Addressing disruptions in childhood routine immunisation services during the COVID-19 pandemic: perspectives from Nepal, Senegal and Liberia. *Bmj Glob Health* 2021;6(7) doi: ARTN e005031

For peer review only

49. Chinele J. Tackling Malawi's fears of routine immunisation: "Children should still be immunised amid the pandemic": Gavi: The Vaccine Alliance; 2021 [updated 24 June 2021. Available from: https://www.gavi.org/vaccineswork/tackling-malawis-fears-routine-immunisation-children-should-still-be-immunised-amid.
50. World Health Organization, UNICEF. Malawi: WHO and UNICEF estimates of immunization coverage: 2020 revision, 2021.
51. World Health Organization. Leave no one behind : guidance for planning and implementing catch-up vaccination, 2021.
52. Peters MA, Mohan D, Naphini P, et al. Linking household surveys and facility assessments: a comparison of geospatial methods using nationally representative data from Malawi. *Population health metrics* 2020;18(1):1-11.

For peer review only

Appendix Table 1. Additional study sample characteristics, children aged 12-23 months in rural areas, Malawi DHS 2015-16.

	Unweighted N	Weighted %
Total	*2740*	*100.0%*
**Individual child indicators**		
Sex of child		
Male	1362	49.1%
Female	1378	50.9%
Birth order		
1st	673	25.3%
2nd	522	18.9%
3rd	453	16.1%
4th	375	13.2%
5th or more	717	26.5%
**Maternal/household indicators**		
Mother's age (Weighted Mean / SD)	*27.2*	*6.6*
Mother's education		
None	331	13.2%
Primary	1963	71.7%
Secondary or higher	446	15.2%
Household wealth index quintile*		
Poorest	769	29.9%
Poorer	678	24.8%
Middle	601	22.3%
Richer	464	16.1%
Richest	228	7.0%
Region of Malawi		
Northern	508	12.0%
Central	959	42.0%
Southern	1273	46.0%

For peer review only

Appendix Table 2. Unadjusted and adjusted comparisons of immunization rates among children in rural areas aged 12-23 months by presence of vaccine-providing facility in 8km radius, Malawi 2013-14.

	Unadjusted rates			Unadjusted logistic regression		Adjusted logistic regression	
	No vaccine-providing facility proximal	Vaccine-providing facility proximal	p-value	OR[1]	95% CI	AOR[1,2]	95% CI
Specific immunization dose coverage							
BCG	96.4%	97.8%	0.22	1.59	[0.71,3.58]	1.54	[0.70,3.39]
Rotavirus 2 doses	87.0%	91.9%	0.02	1.66*	[1.03,2.65]	1.53	[0.95,2.45]
OPV 3 doses	80.4%	81.3%	0.75	1.14	[0.78,1.67]	1.14	[0.80,1.63]
Pentavalent 3 doses	90.7%	93.3%	0.25	1.55	[0.95,2.83]	1.44	[0.81,2.56]
Pneumococcal 3 doses	87.2%	89.4%	0.54	1.33	[0.67,2.64]	1.20	[0.64,2.26]
Measles 1+ dose	86.0%	92.0%	0.03	2.04*	[1.16,3.60]	1.88*	[1.06,3.33]
Coverage of group of immunizations							
All basic vaccines[3]	69.1%	76.6%	0.08	1.57*	[1.02,2.43]	1.54*	[1.02,2.32]
All recommended vaccines[4]	36.7%	53.2%	<0.001	1.89**	[1.28,2.81]	1.97***	[1.35,2.89]
Negative immunization outcomes							
Pentavalent zero dose	5.4%	2.3%	0.03	0.37*	[0.16,0.88]	0.38*	[0.18,0.82]
Pentavalent dropout[5]	4.1%	4.6%	0.75	1.02	[0.51,2.01]	1.10	[0.54,2.24]

[1]Reference is children not proximal to a vaccine-providing facility
[2]Models also control for household wealth, mother's education, mother's age, child sex, child birth order, region of country
[3]Defined as BCG 1 dose, OPV 3 doses, DTP/HBV/Hib (pentavalent) 3 doses, measles 1 dose
[4]Basic + OPV birth dose + rotavirus 2 doses + PCV13 3 doses
[5]Dropout denominator is children with at least 1 dose of pentavalent
* p<0.05, ** p<0.01, *** p<0.001

For peer review only

Appendix Table 3a. Logistic regression models of individual-level vaccination among children 12-23 months living in rural areas who are within 5km of a facility providing vaccination. Only facility-characteristic coefficients reported.[1]

	Specific immunization dose coverage											
	BCG		Rotavirus 2 dose		Polio 3 dose		Pentavalent 3 dose		Pneumococcal 3 dose		Measles 1+ dose	
	AOR	95% CI	AOR	95% CI	AOR	95% CI	AOR	95% CI	AOR	95% CI	AOR	95% CI
Facility type offering vaccination [highest available if >1]												
Hospital	*Ref*	*Ref*	*Ref*	*Ref*	*Ref*	*Ref*	*Ref*	*Ref*	*Ref*	*Ref*	*Ref*	*Ref*
Health centre	0.92	[0.34,2.49]	1.03	[0.54,1.95]	1.07	[0.72,1.58]	0.89	[0.48,1.64]	0.69	[0.40,1.20]	1.17	[0.51,2.64]
Health post/clinic/dispensary	1.88	[0.42,8.48]	0.93	[0.38,2.28]	1.94	[0.97,3.86]	0.84	[0.33,2.16]	0.87	[0.34,2.24]	0.79	[0.27,2.34]
Managing authority of facility/ies offering vaccination												
Government												
No	*Ref*	*Ref*	*Ref*	*Ref*	*Ref*	*Ref*	*Ref*	*Ref*	*Ref*	*Ref*	*Ref*	*Ref*
Yes	2.06	[0.51,8.36]	1.65	[0.89,3.07]	2.32**	[1.37,3.93]	1.76	[0.91,3.42]	1.18	[0.64,2.15]	1.34	[0.70,2.55]
CHAM												
No	*Ref*	*Ref*	*Ref*	*Ref*	*Ref*	*Ref*	*Ref*	*Ref*	*Ref*	*Ref*	*Ref*	*Ref*
Yes	1.71	[0.44,6.67]	1.54	[0.80,2.97]	2.47***	[1.46,4.18]	2.04*	[1.06,3.93]	1.05	[0.60,1.86]	1.39	[0.63,3.07]
Private for-profit												
No	*Ref*	*Ref*	*Ref*	*Ref*	*Ref*	*Ref*	*Ref*	*Ref*	*Ref*	*Ref*	*Ref*	*Ref*
Yes	2.85	[0.31,26.02]	9.83*	[1.20,80.47]	0.82	[0.38,1.79]	5.36	[0.95,30.14]	1.96	[0.33,11.49]	1.57	[0.32,7.73]
NGO												
No	*Ref*	*Ref*	*Ref*	*Ref*	*Ref*	*Ref*	*Ref*	*Ref*	*Ref*	*Ref*	*Ref*	*Ref*
Yes	0.53	[0.13,2.18]	1.42	[0.42,4.81]	0.57	[0.26,1.28]	2.14	[0.54,8.44]	0.76	[0.27,2.14]	1.59	[0.46,5.48]

[1]Models also control for household wealth, education, mother's age, child sex, child birth order, region of country
* p<0.05, ** p<0.01, *** p<0.001

For peer review only

Appendix Table 3b. Logistic regression models of individual-level vaccination among children 12-23 months living in rural areas who are within 5km of a facility providing vaccination. Only facility-characteristic coefficients reported.[1]

	Coverage of group of immunizations				***Negative immunization outcomes***			
	**All basic vaccines[2]**		**All recommended vaccines[3]**		**Pentavalent 0 dose**		**Pentavalent dropout**	
	AOR	95% CI	AOR	95% CI	AOR	95% CI	AOR	95% CI
Facility type offering vaccination [highest available if >1]								
Hospital	*Ref*	*Ref*	*Ref*	*Ref*	*Ref*	*Ref*	*Ref*	*Ref*
Health centre	1.11	[0.75,1.66]	0.79	[0.55,1.14]	1.46	[0.42,5.12]	0.95	[0.49,1.86]
Health post/clinic/dispensary	1.45	[0.75,2.81]	0.93	[0.56,1.54]	0.20	[0.03,1.28]	1.53	[0.57,4.09]
Managing authority of facility/ies offering vaccination								
Government								
No	*Ref*	*Ref*	*Ref*	*Ref*	*Ref*	*Ref*	*Ref*	*Ref*
Yes	1.82*	[1.15,2.88]	1.94**	[1.24,3.03]	0.08***	[0.02,0.33]	0.97	[0.44,2.11]
CHAM								
No	*Ref*	*Ref*	*Ref*	*Ref*	*Ref*	*Ref*	*Ref*	*Ref*
Yes	2.29***	[1.44,3.63]	1.33	[0.86,2.07]	0.11**	[0.02,0.50]	0.64	[0.31,1.32]
Private for-profit								
No	*Ref*	*Ref*	*Ref*	*Ref*	--	--	*Ref*	*Ref*
Yes	0.84	[0.39,1.78]	1.04	[0.48,2.25]	--	--	0.26	[0.04,1.54]
NGO								
No	*Ref*	*Ref*	*Ref*	*Ref*	--	--	*Ref*	*Ref*
Yes	0.60	[0.28,1.28]	0.62	[0.35,1.10]	--	--	0.60	[0.15,2.32]

[1]Models also control for household wealth, education, mother's age, child sex, child birth order, region of country
[2]Defined as BCG 1 dose, OPV 3 dose, DTP/HBV/Hib (pentavalent) 3 dose, measles 1 dose
[3]Basic + OPV birth dose + rotavirus 2 doses + PCV13 3 doses
* p<0.05, ** p<0.01, *** p<0.001

For peer review only

STROBE Statement—Checklist of items that should be included in reports of ***cross-sectional studies***

	Item No	Recommendation	Page No
**Title and abstract**	1	(*a*) Indicate the study's design with a commonly used term in the title or the abstract	1
		(*b*) Provide in the abstract an informative and balanced summary of what was done and what was found	2
**Introduction**			
Background/rationale	2	Explain the scientific background and rationale for the investigation being reported	4-5
Objectives	3	State specific objectives, including any prespecified hypotheses	5
**Methods**			
Study design	4	Present key elements of study design early in the paper	5-6
Setting	5	Describe the setting, locations, and relevant dates, including periods of recruitment, exposure, follow-up, and data collection	5-6
Participants	6	(*a*) Give the eligibility criteria, and the sources and methods of selection of participants	6
Variables	7	Clearly define all outcomes, exposures, predictors, potential confounders, and effect modifiers. Give diagnostic criteria, if applicable	7
Data sources/ measurement	8*	For each variable of interest, give sources of data and details of methods of assessment (measurement). Describe comparability of assessment methods if there is more than one group	7
Bias	9	Describe any efforts to address potential sources of bias	8, 15
Study size	10	Explain how the study size was arrived at	6, 9
Quantitative variables	11	Explain how quantitative variables were handled in the analyses. If applicable, describe which groupings were chosen and why	7
Statistical methods	12	(*a*) Describe all statistical methods, including those used to control for confounding	7-8
		(*b*) Describe any methods used to examine subgroups and interactions	8
		(*c*) Explain how missing data were addressed	7
		(*d*) If applicable, describe analytical methods taking account of sampling strategy	8
		(*e*) Describe any sensitivity analyses	8
**Results**			
Participants	13*	(a) Report numbers of individuals at each stage of study—eg numbers potentially eligible, examined for eligibility, confirmed eligible, included in the study, completing follow-up, and analysed	9
		(b) Give reasons for non-participation at each stage	N/A
		(c) Consider use of a flow diagram	N/A
Descriptive data	14*	(a) Give characteristics of study participants (eg demographic, clinical, social) and information on exposures and potential confounders	9-10
		(b) Indicate number of participants with missing data for each variable of interest	7
Outcome data	15*	Report numbers of outcome events or summary measures	8-9
Main results	16	(*a*) Give unadjusted estimates and, if applicable, confounder-adjusted estimates and their precision (eg, 95% confidence interval). Make clear which confounders were adjusted for and why they were included	10-12

For peer review only - http://bmjopen.bmj.com/site/about/guidelines.xhtml

		(*b*) Report category boundaries when continuous variables were categorized	7
		(*c*) If relevant, consider translating estimates of relative risk into absolute risk for a meaningful time period	N/A
Other analyses	17	Report other analyses done—eg analyses of subgroups and interactions, and sensitivity analyses	13
**Discussion**			
Key results	18	Summarise key results with reference to study objectives	3-14
Limitations	19	Discuss limitations of the study, taking into account sources of potential bias or imprecision. Discuss both direction and magnitude of any potential bias	15
Interpretation	20	Give a cautious overall interpretation of results considering objectives, limitations, multiplicity of analyses, results from similar studies, and other relevant evidence	14-15
Generalisability	21	Discuss the generalisability (external validity) of the study results	14-15
**Other information**			
Funding	22	Give the source of funding and the role of the funders for the present study and, if applicable, for the original study on which the present article is based	13

*Give information separately for exposed and unexposed groups.

Note: An Explanation and Elaboration article discusses each checklist item and gives methodological background and published examples of transparent reporting. The STROBE checklist is best used in conjunction with this article (freely available on the Web sites of PLoS Medicine at http://www.plosmedicine.org/, Annals of Internal Medicine at http://www.annals.org/, and Epidemiology at http://www.epidem.com/). Information on the STROBE Initiative is available at www.strobe-statement.org.

For peer review only

BMJ Open

The association between childhood immunization coverage and proximity to health facilities in rural settings: A cross-sectional analysis of Service Provision Assessment 2013-14 facility data and Demographic and Health Survey 2015-16 individual data in Malawi

Journal:	*BMJ Open*
Manuscript ID	bmjopen-2022-061346.R2
Article Type:	Original research
Date Submitted by the Author:	12-May-2022
Complete List of Authors:	Johns, Nicole; World Health Organization, Department of Data and Analytics<br>Hosseinpoor, Ahmad Reza; World Health Organization, Department of Data and Analytics<br>Chisema, Mike; Malawi Ministry of Health<br>Danovaro-Holliday, M Carolina; World Health Organization, Department of Immunization, Vaccines and Biologicals<br>Kirkby, Katherine; World Health Organization, Department of Data and Analytics<br>Schlotheuber, Anne; World Health Organization, Department of Data and Analytics<br>Shibeshi, Messeret; World Health Organization, Inter-Country Support Team for East and Southern Africa<br>Sodha, Samir V.; World Health Organization, Department of Immunization, Vaccines and Biologicals<br>Zimba, Boston; World Health Organization, Malawi Country Office
**Primary Subject Heading**:	Global health
Secondary Subject Heading:	Public health, Paediatrics
Keywords:	PUBLIC HEALTH, Community child health < PAEDIATRICS, Paediatric infectious disease & immunisation < PAEDIATRICS

SCHOLARONE™
Manuscripts

I, the Submitting Author has the right to grant and does grant on behalf of all authors of the Work (as defined in the below author licence), an exclusive licence and/or a non-exclusive licence for contributions from authors who are: i) UK Crown employees; ii) where BMJ has agreed a CC-BY licence shall apply, and/or iii) in accordance with the terms applicable for US Federal Government officers or employees acting as part of their official duties; on a worldwide, perpetual, irrevocable, royalty-free basis to BMJ Publishing Group Ltd ("BMJ") its licensees and where the relevant Journal is co-owned by BMJ to the co-owners of the Journal, to publish the Work in this journal and any other BMJ products and to exploit all rights, as set out in our licence.

The Submitting Author accepts and understands that any supply made under these terms is made by BMJ to the Submitting Author unless you are acting as an employee on behalf of your employer or a postgraduate student of an affiliated institution which is paying any applicable article publishing charge ("APC") for Open Access articles. Where the Submitting Author wishes to make the Work available on an Open Access basis (and intends to pay the relevant APC), the terms of reuse of such Open Access shall be governed by a Creative Commons licence – details of these licences and which Creative Commons licence will apply to this Work are set out in our licence referred to above.

Other than as permitted in any relevant BMJ Author's Self Archiving Policies, I confirm this Work has not been accepted for publication elsewhere, is not being considered for publication elsewhere and does not duplicate material already published. I confirm all authors consent to publication of this Work and authorise the granting of this licence.

For peer review only

The association between childhood immunization coverage and proximity to health facilities in rural settings: A cross-sectional analysis of Service Provision Assessment 2013-14 facility data and Demographic and Health Survey 2015-16 individual data in Malawi

Nicole E. Johns[1], Ahmad Reza Hosseinpoor[1*], Mike Chisema[2], M. Carolina Danovaro-Holliday[3], Katherine Kirkby,[1] Anne Schlotheuber,[1] Messeret Shibeshi,[4] Samir V. Sodha,[3] Boston Zimba[5]

[1]Department of Data and Analytics, World Health Organization, Geneva, Switzerland.
[2]Malawi Ministry of Health, Lilongwe, Malawi.
[3]Department of Immunization, Vaccines and Biologicals, World Health Organization, Geneva, Switzerland.
[4]Inter-Country Support Team for East and Southern Africa, World Health Organization, Harare, Zimbabwe
[5]Malawi Country Office, World Health Organization, Lilongwe, Malawi
*Corresponding author

Corresponding Author:
Ahmad Reza Hosseinpoor
hosseinpoora@who.int
Department of Data and Analytics
Division of Data, Analytics and Delivery for Impact
World Health Organization
20, Avenue Appia, CH-1211 Geneva 27, Switzerland

Manuscript word count: 3987

Key words: Childhood immunization; vaccination; equity; rural; Malawi

For peer review only

ABSTRACT

Objectives: Despite significant progress in childhood vaccination coverage globally, substantial inequality remains. Remote rural populations are recognized as a priority group for immunization service equity. We aimed to link facility and individual data to examine the relationship between distance to services and immunization coverage empirically, specifically using a rural population.

Design & setting: Retrospective cross-sectional analysis of facility data from the 2013-14 Malawi Service Provision Assessment and individual data from the 2015-16 Malawi Demographic and Health Survey, linking children to facilities within a 5-kilometer radius. We examined associations between proximity to health facilities and vaccination receipt via bivariate comparisons and logistic regression models.

Participants: 2740 children aged 12-23 months living in rural areas.

Outcome measures: Immunization coverage for the six vaccines included in the Malawi EPI schedule for children under one at time of study, as well as two composite vaccination indicators (receipt of basic vaccines and receipt of all recommended vaccines), zero-dose pentavalent coverage, and pentavalent dropout.

Findings: 72% (706/977) of facilities offered childhood vaccination services. Among children in rural areas, 61% were proximal to (within 5km) a vaccine-providing facility. Proximity to a vaccine-providing health facility was associated with increased likelihood of having received the rotavirus vaccine (93% vs 88%, p=0.004) and measles vaccine (93% vs 89%, p=0.01) in bivariate tests. In adjusted comparisons, how close a child was to a health facility remained meaningfully associated with how likely they were to have received rotavirus vaccine (AOR 1.63, 95% CI 1.12-2.33) and measles vaccine (AOR 1.62, 95% CI 1.11-2.37).

Conclusion: Proximity to health facilities was significantly associated with likelihood of receipt for some, but not all, vaccines. Our findings reiterate the vulnerability of children residing far from static vaccination services; efforts that specifically target remote rural populations living far from health facilities are warranted to ensure equitable vaccination coverage.

For peer review only

STRENGTHS AND LIMITATIONS OF THE STUDY

- Health facility and individual data were linked to examine immunization service access and equality for rural populations using a nationally-representative dataset.
- Straight-line distance to nearest immunization service was calculated, and the association between proximity to a facility providing services and child-level immunization coverage outcomes were examined.
- Analyses accounted for additional known predictors of immunization coverage such as birth order and household wealth.
- Straight-line distance to nearest services does not reflect actual access, which is tied to road networks, geography, seasonality, vehicle access, and preferred facilities.
- Distance is only one of many factors which render a population hard-to-reach or hard-to-vaccinate.

For peer review only

INTRODUCTION

Despite significant progress in childhood vaccination coverage globally since the establishment of the Expanded Programme on Immunization (EPI) in 1974, substantial inequality in vaccination coverage remains.[1-4] Within countries, immunization coverage tends to be lowest among the most disadvantaged subgroups, including those living in rural areas.[2 5 6] The Equity Reference Group for Immunization has identified remote rural populations (those living furthest from population centers) as one of the most pressing priority areas of work for improving immunization equity.[7 8] The Immunization Agenda 2030 (IA2030) recognizes coverage and equity as strategic priorities and has the stated goal to leave no one behind, i.e. to ensure that hard to reach and marginalized populations are included and centered in immunization policies and initiatives, and to ultimately achieve universal immunization coverage.[9 10] Similarly, Gavi's most recent strategy also centers the theme of leaving no one behind and indicates equity as an organizing principle and key goal.[11] Globally, governments and key stakeholders in immunization are recognizing equity, including reaching remote rural populations, as a central priority.[8]

Immunization services for remote rural populations involve a number of barriers. On the side of the procurement, distribution, and provision of vaccines, barriers include increased marginal costs of administration, challenges of sufficient health worker availability and training, physical access for supply delivery, and cold-chain continuity.[7] One of the largest barriers on the side of the remote rural patient to immunization provision is physical access to static or outreach services. Distance to a health facility is a commonly cited barrier to vaccination in nationally representative studies and systematic reviews.[12-15] Several studies have examined distance empirically, and find significant negative associations between distance to vaccination site and likelihood of vaccination, though all rely on subnational samples.[16-18] One nationally-representative study from Nigeria found that distance to a health facility (whether it provided vaccination services or not) was significantly and negatively associated with receipt of most vaccines.[19] Distance to a health facility that routinely provides immunization services is likely associated with immunization coverage, particularly among rural populations.

The Malawi health system consists of public, private for profit, and private not for profit sectors; the majority of healthcare services are provided by the government, followed by the major religious provider Christian Health Association of Malawi (CHAM).[20] Though vaccination services may be available at all facilities, the majority of children receive vaccinations through government facilities or outreach clinics.[21] In the community, immunization services are most frequently provided by health workers known as health surveillance assistants (HSAs), who provide door-to-door visitations and staff village, outreach, and mobile clinics.[20 22] Routine

immunization services are also supported by other periodic supplementary immunization activities (SIAs).[23-25]

As of the 2015-16 Malawi Demographic and Health Survey (DHS), an estimated 76% (95% CI 74%-78%) of children age 12-23 months had received basic immunizations (defined as one dose of Bacillus Calmette-Guérin (BCG); three doses of oral polio vaccine (OPV); three doses of pentavalent vaccine (diphtheria, tetanus, pertussis [DTP], hepatitis B virus [HBV], and *Haemophilus influenzae* type b [Hib]); and one dose of measles vaccine), a decline from 81% (95% CI 79-83%) in the 2010-11 DHS.[26] Contrary to findings in other sub-Saharan settings and prior research within Malawi, childhood vaccination coverage was in fact higher among children living in rural areas (77%, 95% CI 74%-79%) than children living in urban areas (70%, 95% CI 63%-76%).[12 26] However, such figures conceal inequalities in access to immunization *within* rural populations, where the most remote are still likely under-immunized.[7] Data from the 2015-16 Malawi DHS confirms these within-rural inequalities: coverage of basic vaccines varied by more than 25 percentage points across rural strata, from 65% (95% CI 53%-75%) to 91% (95% CI 84%-95%).[27] Understanding inequities within rural populations is particularly relevant in Malawi, where 84% of the population live in rural areas.[28]

The policy of the Malawi Ministry of Health is that all Malawians should live within 8 kilometers (km) of a public health facility, though as of 2014 more than two million people were estimated to live further than this from care.[29] The 8km policy is intended to ensure “reasonable walking distance” to healthcare, which is particularly relevant given low vehicle ownership (only 4% of households in rural areas had a car, truck, motorcycle or scooter as of the 2015-16 DHS).[27] Several studies from Malawi suggest that residing far from a facility providing vaccination services is a driver of inequitable immunization coverage for remote rural populations.[30 31] Research has documented the relationship between empirically-derived distance to facility and other healthcare services and outcomes in Malawi;[32-37] to our knowledge, no other study has examined empirically-derived distance to facilities providing vaccination services and immunization coverage in Malawi.

In this study, we hypothesize that children living in rural areas aged 12-23 months who are proximal to a vaccine-providing health facility will be significantly more likely to have received immunizations than children who live further than 5km from the nearest vaccine-providing facility. Substantial research has explored individual-level factors associated with vaccination, but less work has demonstrated an association between empirically-derived facility access and immunization coverage, particularly using nationally-representative data. The nature of the data available in Malawi pose a unique opportunity to explore this quantitatively.

METHODS

Data sources

Facility data come from the Malawi 2013-14 DHS Service Provision Assessment (SPA) survey, a census of formal-sector public and private health facilities in Malawi.[38] It includes all static sites but not outreach clinic locations. Facilities included in the survey were hospitals, health centres, dispensaries, clinics, and health posts; managing authorities included government, CHAM and other faith-based organizations, private sector for-profit organizations, non-governmental organizations (NGOs), and companies.

We used data from the Malawi 2015-16 DHS, the most recent available DHS survey in the country and the closest in time to the SPA, to determine immunization coverage among children aged 12-23 months residing in rural areas. Vaccine doses included were those six in the Malawi EPI schedule for children under one at the time of data collection: BCG, OPV, pentavalent, pneumococcal conjugate vaccine 13-valent (PCV13), rotavirus vaccine, and measles-containing vaccine (Table 1).[39] Details regarding DHS sampling, implementation, and content are published elsewhere.[26] Individual, family, and community characteristics were also extracted from this dataset.

Table 1. Malawi EPI vaccination schedule for children under one year, 2015[1]

Age	Vaccine
Birth up to two weeks after birth	BCG<br>Birth dose OPV
6 weeks	OPV dose 1<br>Pentavalent dose 1<br>PCV13 dose 1<br>Rotavirus dose 1
10 weeks	OPV dose 2<br>Pentavalent dose 2<br>PCV13 dose 2<br>Rotavirus dose 2
14 weeks	OPV dose 3<br>Pentavalent dose 3<br>PCV13 dose 3
9 months	Measles dose 1

[1]Though this schedule reflects the EPI schedule at time of data analyzed here, several changes to the EPI have gone into effect since 2015. The measles vaccine was replaced with measles and rubella vaccine in July 2017. The trivalent OPV was replaced with bivalent OPV in 2016, and inactivated polio vaccine (IPV) was introduced starting in December 2018. The malaria vaccine (RTS,S/ASO1) was also piloted in 2019 but is not yet in the national EPI vaccination schedule as of May 2022.

For peer review only - http://bmjopen.bmj.com/site/about/guidelines.xhtml

Geographic data linkage

Facility geo-location, provided as part of SPA data, was matched to individual data using the DHS-suggested technique of a Euclidian (straight-line) distance buffer around each DHS cluster centroid.[40] This buffer is defined as 5km in rural areas; this corresponds with the DHS offset of cluster GPS locations by up to 5km in rural areas. Therefore, we defined proximity to a vaccine-providing facility as children living in rural areas within 5km of a facility that reported regularly providing vaccination services on-site and/or via outreach. We also tested alternate buffer distances, including 8km based on Malawi Ministry of Health definitions (see sensitivity analysis description below).[29]

Measures

Facility data indicated vaccination provision, stock, fees, and preparedness. For the sake of these analyses, only presence of a health facility offering vaccination services was used. Indicators of vaccination fees were not used because very few facilities (2%) reported charging vaccination-specific fees. Indicators of full EPI provision were not used because most facilities offering any vaccination services offered all EPI vaccines (99% of facilities offering vaccines offered full EPI). Despite a 2-3 year gap between the survey of facilities (2013-14) and survey of individuals (2015-16, children born October 2013-February 2015), retroactive reporting of immunization for children 12-23 months reflects immunization receipt close to the time of facility survey, and provision of any vaccination services is likely to have remained constant over that time.

Immunization coverage was defined as the proportion of children aged 12–23 months who received the indicated vaccine dose by the time of survey. For OPV, pentavalent, and PCV13 vaccines, children were considered having received the complete series of each vaccine type if they had three doses. Birth dose OPV was considered separately from the three-dose OPV series. For rotavirus vaccine, two doses were considered the complete series, and for BCG and measles, one dose (or more) was considered complete. We also examined receipt of basic vaccines, also termed full immunization with basic vaccine doses, (one dose BCG, three doses OPV, three doses pentavalent, and one dose measles) as well as receipt of all recommended vaccines (basic vaccine doses plus birth dose OPV, two doses rotavirus, and three doses PCV13). Finally, we created indicators of zero-dose pentavalent coverage (no receipt of pentavalent vaccine) and pentavalent dropout (receipt of the first dose, but not the third dose, of pentavalent vaccine). For all vaccines, both mother’s recall and vaccination card verification (with or without date) were used to determine coverage. Similarly, vaccines received within the recommended time frame and any time after were included. The few ‘Don’t know’ responses

were coded as non-receipt of the corresponding vaccine/dose (<1% for all, N=0 to 10 per vaccine dose).

Analyses

All analyses were limited to rural populations, as defined by DHS.[41] We first used bivariate Pearson's chi-squared tests to assess associations between vaccination coverage and proximity to a vaccine-providing health facility among children living in rural areas, for all immunization outcomes. We then constructed unadjusted and adjusted logistic regression models to assess the association between proximity to a vaccine-providing health facility and immunization outcomes among children living in rural areas, with adjusted models controlling for: child's sex [male, female], birth order [1, 2, 3, 4, 5+], household wealth [quintile], mother's age [years], mother's education [none, primary, secondary or higher], and subnational region [Northern, Central, Southern]. All covariates were selected *a priori* based on established association with likelihood of immunization and availability in DHS survey data.[21 31 42]

We then conducted several sensitivity analyses. First, we constructed multinomial models to separate immunization verified by vaccine card and immunization noted via mother's recall, to examine whether the association between facility proximity and immunization was sensitive to the nature of vaccination ascertainment. For each vaccine, we defined a 3-level outcome as: not received (referent group), received and verified by vaccine card, and received as noted via mother's recall. Second, we modified to the proximity radius to 8km rather than the 5km suggested by DHS, to align with Malawi Ministry of Health policy and assess whether findings were sensitive to the distance radius used.[29 40] Third, we restricted the sample to children residing within 5km of a vaccine-providing facility, and examined whether facility level of care and managing authority were associated with vaccination coverage in adjusted logistic regression models. We captured facility level of care via a single variable, classified as: hospital, health centre, or health post/clinic/dispensary. We assigned children who were within 5km of multiple facilities providing vaccines to the highest level of care among those proximal facilities. We captured facility managing authority via a set of four indicators, separately assessing whether a child was proximal to a vaccine-providing facility managed by the government, CHAM, private for-profit, and/or NGO. Fourth, as a final post-hoc analysis, we examined vaccine stock at time of SPA survey, considering a vaccine in stock if it was observed, or reported to be available, and was not expired. For each of the six examined vaccines, we replicated adjusted analyses of immunization coverage for the total sample using an indicator of the corresponding vaccine stock within 5km, and the same analyses limited to children within 5km of a vaccine-providing facility.

All individual-level analyses accounted for complex sampling design using provided DHS survey weights. Statistical significance was set at $p<0.05$ for Pearson's chi-squared tests, Adjusted Odds Ratios (AORs), and adjusted Relative Risk Ratios (aRRRs); 95% confidence intervals (CIs) are reported throughout. We conducted analyses using ArcGIS Pro 2.8.0 and STATA 15.1.

Ethics Approval

Ethical approval for data collection was obtained by the DHS Program and implementing partners at time of survey, and participants provided informed consent to participate in the DHS survey at the time of data collection. This secondary data analysis using publicly-available de-identified data was reviewed and approved by the National Health Sciences Research Committee of Malawi (#20220106).

Patient and public involvement

Due to the nature of this analysis, patients and the public were not involved in the design, conduct, reporting, or dissemination of this research.

RESULTS

Of 977 facilities surveyed, 72% (N=706) offered any childhood immunization services (Table 2).

Table 2. Health facilities offering childhood immunization services, Malawi SPA 2013-14.

	N	%
Total	*706*	*100.0%*
Type of vaccination offerings		
% offering all EPI vaccines	696	98.6%
Facility type		
Central hospital	1	0.1%
District hospital	24	3.4%
Rural/community hospital	41	5.8%
Other hospital	31	4.4%
Health centre	465	65.9%
Maternity	3	0.4%
Dispensary	40	5.7%
Clinic	81	11.5%
Health post	20	2.8%
Managing authority		
Government/public	457	64.7%
Christian Health Association of Malawi (CHAM)	153	21.7%
Private for profit	39	5.5%
Other mission/faith-based	6	0.9%

For peer review only

Non-governmental organization (NGO)	19	2.7%
Company	32	4.5%

Individual survey data included 2740 children aged 12-23 months living in rural areas (Appendix Table 1). All living children aged 12-23 months residing in rural areas were included. The majority of children were proximal to health facilities: 64% were within 5km of *any* health facility and 61% were within 5km of a vaccine-providing facility (Table 3). Proximity was often limited to a single facility; only 5% were within 5km of two or more vaccine-providing facilities.

Overall, immunization coverage among children in rural areas was above 80% for all examined individual vaccines: 97.5% 1-dose BCG, 82.2% 3-dose OPV, 93.4% 3-dose pentavalent, 91.1% 2-dose rotavirus, 89.7% 3-dose PCV13, and 91.7% 1-dose measles (Table 3). However, full immunization coverage was lower: three-fourths (76.8%) of children had basic vaccine doses and only half (50.4%) had all vaccines recommended for children under one. Few children received no doses of pentavalent vaccine (2.5%), and among children who started their pentavalent vaccine series, 4.2% did not complete it (indicating dropout). Among children with vaccination date information available, all vaccines except measles had 99% or greater receipt within the first year of life, while 93.4% of measles first doses were received before age one (results not shown).

Table 3. Study sample - children aged 12-23 months in rural areas, Malawi DHS 2015-16.

	Unweighted N	Weighted %
Total	*2740*	*100.0%*
**Facility proximity indicators**		
Facility proximity within 5km		
No facility within 5km	978	36.3%
Any facility within 5km	*1762*	*63.7%*
Vaccine-providing facility within 5km	1698	61.2%
Non-vaccine-providing facility only within 5km	64	2.6%
Proximal to vaccine-providing facility, by type[1]		
Hospital	403	12.1%
Health centre	1264	47.2%
Health post/clinic/dispensary	289	10.9%
Proximal to vaccine-providing facility, by managing authority[1]		
Government/public	1305	46.7%
CHAM	501	16.9%
Private for profit	53	2.8%
NGO	74	2.8%
**Immunization coverage indicators**		
Specific immunization dose coverage		

For peer review only

BCG	2673	97.5%
OPV 3 doses	2273	82.2%
Pentavalent 3 doses	2559	93.4%
Rotavirus 2 doses	2510	91.1%
PCV13 3 doses	2460	89.7%
Measles 1+ dose	2524	91.7%
Coverage of group of immunizations		
Basic vaccines[2]	2129	76.8%
All recommended vaccines[3]	1450	50.4%
Negative immunization outcomes		
Pentavalent zero dose	61	2.5%
Pentavalent dropout[4]	120	4.2%

[1]Children could be proximal to more than one vaccine-providing facility and thus more than one vaccine-providing facility type/managing authority

[2]Defined as BCG 1 dose, OPV 3 doses, DTP/HBV/Hib (pentavalent) 3 doses, measles 1 dose

[3]Basic + OPV birth dose + rotavirus 2 doses + PCV13 3 doses

[4]Dropout denominator is children with at least 1 dose of pentavalent; represents percent of children receiving first but not third dose of pentavalent.

Children living in rural areas who were proximal to a vaccine-providing facility were more likely to have some, but not all, vaccine doses in unadjusted bivariate tests when compared to children who were not in proximity to a vaccine-providing facility: rotavirus (93% vs 88%, p=0.004), measles (93% vs 89%, p=0.01), and full immunization with basic vaccines (79% vs 74%, p=0.04) and with all recommended vaccines (54% vs 44%, p<0.001) (Table 4). No significant bivariate differences were observed for BCG, OPV, pentavalent, or PCV13 vaccine, nor pentavalent zero dose or dropout.

These relationships hold true in fully adjusted regression models (Table 4). Compared to children living greater than 5km from the nearest facility providing immunization services, children living proximal to a vaccine-providing facility had greater odds of receiving rotavirus (AOR 1.63, 95% CI 1.12-2.33) and measles (AOR 1.62, 95% CI 1.11-2.37) vaccines. They also had marginally greater odds of receiving basic vaccines (AOR 1.28, 95% CI 0.99-1.65, p=0.057) and greater odds of receiving all recommended vaccines (AOR 1.38, 95% CI 1.09-1.74). They had somewhat lower odds of being zero-dose for pentavalent (AOR 0.53, 95% CI 0.28-1.01, p=0.052). No statistically significant associations were observed with BCG, OPV, pentavalent, or PCV13 receipt, nor pentavalent dropout.

Table 4. Unadjusted and adjusted comparisons of immunization rates among children in rural areas aged 12-23 months by presence of vaccine-providing facility in 5km radius, Malawi 2013-14.

	**Unadjusted rates**			**Unadjusted logistic regression**		**Adjusted logistic regression**		**Adjusted multinomial logistic regression, three-level recording-method-specific outcome**			
								Mother recall		Vaccine card	
	No vaccine-providing facility proximal	Vaccine-providing facility proximal	p-value	OR[1]	95% CI	AOR[1,2]	95% CI	aRRR[1,2]	95% CI	aRRR[1,2]	95% CI
Specific immunization dose coverage											
BCG	97.4%	97.6%	0.78	1.09	[0.58,2.05]	1.17	[0.63,2.20]	1.28	[0.67,2.42]	1.15	[0.61,2.16]
Rotavirus 2 doses	88.5%	92.8%	0.004	1.68**	[1.17,2.41]	1.63**	[1.13,2.33]	1.71*	[1.12,2.60]	1.72**	[1.18,2.51]
OPV 3 doses	81.4%	82.6%	0.55	1.09	[0.83,1.42]	1.08	[0.83,1.40]	1.15	[0.75,1.75]	1.11	[0.85,1.46]
Pentavalent 3 doses	92.6%	94.0%	0.30	1.24	[0.83,1.87]	1.23	[0.84,1.82]	1.33	[0.85,2.09]	1.20	[0.80,1.79]
PCV13 3 doses	88.9%	90.2%	0.49	1.15	[0.78,1.68]	1.07	[0.75,1.53]	1.09	[0.70,1.68]	1.1	[0.76,1.59]
Measles 1+ dose	89.3%	93.3%	0.01	1.68**	[1.14,2.48]	1.62*	[1.11,2.37]	1.52*	[1.04,2.24]	1.59*	[1.07,2.35]
Coverage of group of immunizations											
Basic vaccines[3]	73.8%	78.7%	0.04	1.31*	[1.01,1.69]	1.28	[0.99,1.65]	--	--	--	--
All recommended vaccines[4]	45.0%	53.7%	<0.001	1.42**	[1.13,1.78]	1.38**	[1.09,1.74]	--	--	--	--
Negative immunization outcomes											
Pentavalent zero dose	3.4%	1.9%	0.08	0.55	[0.28,1.10]	0.53	[0.28,1.01]	--	--	--	--
Pentavalent dropout[5]	4.1%	4.2%	0.91	1.02	[0.67,1.56]	1.05	[0.69,1.61]	--	--	--	--

[1]Reference is children not proximal to a vaccine-providing facility
[2]Models also control for household wealth, mother's education, mother's age, child sex, child birth order, region of country
[3]Defined as BCG 1 dose, OPV 3 doses, DTP/HBV/Hib (pentavalent) 3 doses, measles 1 dose
[4]Basic + OPV birth dose + rotavirus 2 dose + PCV13 3 dose
[5]Dropout denominator is children with at least 1 dose of pentavalent
* p<0.05, ** p<0.01, *** p<0.001

Associations between proximity to a vaccine-providing facility and immunization coverage were similar for both mother's recall and vaccination card documented immunization in multinomial models (Table 4). The majority of children in rural areas (80%, 95% CI 78%-82%) had a vaccination card which was seen. Children were both more likely to have vaccination card-recorded rotavirus vaccination (AOR 1.71, 95% CI 1.12-2.60) or mother-recalled rotavirus vaccination (AOR 1.72, 95% CI 1.18-2.51) if they were proximal to a vaccine-providing facility. Children were also both more likely to have vaccination card recorded measles vaccination (AOR 1.59, 95% CI 1.07-2.35) or mother-recalled measles vaccination (AOR 1.52, 95% CI 1.04-2.24) if they were proximal to a vaccine-providing facility. No significant associations between proximity to vaccine-providing facility and immunization, whether ascertained through mother recall or vaccination card, were observed for BCG, OPV, pentavalent, or PCV13 vaccines.

When considering proximity as 8km rather than 5km, 87% of children were proximal to a vaccine-providing facility; associations with immunization coverage were similar to those with 5km proximity definition (Appendix Table 2). We observed significantly greater immunization coverage among children within 8km of a vaccine-providing facility for rotavirus (91.9% vs 87.0%, p=0.02), measles (92.0% vs 86.0%, p=0.03), and all recommended vaccines (53.2% vs 36.7%, p=0.001) in unadjusted comparisons. Additionally, zero-dose pentavalent receipt was significantly less common among children within 8km of a vaccine-providing facility compared to those not (2.3% vs 5.4%, p=0.03). Findings were similar in adjusted models: measles (AOR 1.88, 95% CI 1.06-3.33, p=0.03), basic vaccines (AOR 1.54, 95% CI 1.02-2.32, p=0.04), all recommended vaccines (AOR 1.97, 95% CI 1.35-2.89, p<0.001), zero-dose pentavalent (AOR 0.38, 95% CI 0.18-0.83, p=0.01). Rotavirus vaccine did not have a significant association with 8km proximity to a vaccine-providing facility in adjusted models (AOR 1.53, 95% CI 0.95-2.45, p=0.08). As with 5km findings, no significant associations were observed for OPV, BCG, pentavalent, or PCV13 vaccines.

When limited to children within 5km of a vaccine-providing facility, the level of the facility was not associated with any of the immunization indicators (Appendix Tables 3a, 3b). Proximity to a government-run facility offering vaccination services was associated with greater odds of receipt of 3-dose OPV (AOR 2.32, 95% CI 1.37-3.93), basic vaccines (AOR 1.82, 95% CI 1.15-2.88), and all recommended vaccines (AOR 1.94, 95% CI 1.24-3.03), and lower odds of zero-dose pentavalent (AOR 0.08, 95% CI 0.02-0.33). Proximity to a CHAM facility offering vaccination services was associated with greater odds of receipt of 3-dose OPV (AOR 2.47, 95% CI 1.46-4.18), 3-dose pentavalent (AOR 2.04, 95% CI 1.05-3.93), and basic vaccines (AOR 2.29, 95% CI 1.44-3.63), and lower odds of zero-dose pentavalent (AOR 0.11, 95% CI 0.02-0.50).

Current vaccine stock was positively and significantly associated with rotavirus (AOR 1.67, 95% CI 1.17-2.39) and measles (AOR 1.52, 95% CI 1.04-2.22) immunization coverage; these findings were consistent to those using indicators of immunization service availability generally (results not shown). When limited to children living within 5km of a vaccine-providing facility, vaccine stock was not associated with immunization coverage for any of the six examined vaccines.

DISCUSSION

Among children aged 12-23 months living in rural areas, proximity to a health facility providing vaccination services was associated with increased likelihood of rotavirus and measles vaccine receipt (and therefore

receipt of all recommended vaccines, as rotavirus and measles are part of this composite indicator), as well as with decreased likelihood of zero-dose pentavalent vaccination. Even when accounting for known child, mother, family, and geographic determinants of immunization, how close a child was to a health facility was meaningfully associated with how likely they were to have received certain vaccines.

Proximity to a vaccine-providing health facility among rural children can be considered a proxy for remote ruralness: those who are far from a vaccine-providing facility are also likely to be far from a population center and far from services more generally. Therefore, these findings indicate that remote rural children in Malawi were likely inequitably under-vaccinated at the time of the 2015-16 DHS. This is an important equity consideration; despite higher immunization coverage amongst rural children than urban children overall, these findings reiterate that rural populations are not a monolith and that inequities are present beyond urban/rural differences. While geographic distance is just one of many factors which render a population hard-to-reach or hard-to-vaccinate, it is relatively easy to define and target, and is a meaningful correlate of coverage.[43] Equity-focused interventions and monitoring efforts should therefore use as granular geographic delineations as possible, with particular focus on those populations furthest from care.

We observed differential associations by type of immunization. The measles vaccine is delivered on its own several months after other infant vaccinations, and the requirement of a specific healthcare visit to obtain it may exacerbate the barrier of increased distance for accessing care. While rotavirus vaccine is offered simultaneously with other vaccinations, it was introduced to the Malawi EPI in October 2012 and outreach efforts may still have been in scale-up at the time the children under consideration were eligible for vaccination. It was introduced with strict age restrictions (first dose at 6-12 weeks, 4 weeks between doses, second dose no later than 16 weeks), which were not formally removed from the Malawi EPI until 2017.[44] The narrow time range for vaccination created by these limits may have made the vaccine harder to access for populations far from facilities. Conversely, the null findings for 3-dose OPV and 3-dose pentavalent vaccine add evidence to the success of the Malawi EPI in ensuring access to these vaccines among rural populations more broadly.[39]

In analyses restricted to children living within 5km of vaccine-providing facilities, we observed no differences in immunization coverage by facility level of care, but we did find significant differences by facility managing authority. Similar odds of immunization regardless of facility level are unsurprising given that immunization services do not require highly specialized equipment or intensive provider training. Greater likelihood of immunization when the proximal facility was managed by the government or CHAM likely reflects the higher rate of outreach efforts performed by these authorities, the lack of fees for immunization at these facilities, and greater resource availability, training, and oversight more broadly.[45] Additional public-sector outreach efforts in areas where the only health facilities are run privately may thus be warranted.

Our finding that immunization coverage is inequitably lower among children further from health facilities is particularly relevant in light of the COVID-19 pandemic and its effects on childhood immunization services. Pandemic effects on childhood immunization services include service provision limitations such as suspension of outreach activities, disruption and suspension of in-facility services, disruption to vaccine and supply availability, shortages of available healthcare workers, and service utilization limitations including travel restrictions, concern for health and safety in seeking services, and lack of knowledge of service availability.[46 47] Mitigation efforts within Malawi and in other country contexts reduced the disruptions to routine

immunization, resulting in only a 1-2% decline in coverage of vaccinations at the national level for 2020.[48-50] However, remote rural patients are most likely to have experienced these disruptions, given the reliance on outreach services or on travel to seek care. Strategies will be needed to ensure that missed children are caught-up for equitable immunization coverage.[51] Practically, these additional efforts should include campaigns and outreach efforts, as these are less resource-intensive to implement than the construction, staffing, and maintenance of new facilities. These outreach efforts can be tailored to reach the most rural populations by inclusion of transportation considerations such as providing cars, motor bikes, and fuel, as well as supplies which can be carried long distances and be used in areas with limited infrastructure. Our findings also add further support to the stated goal of the Malawi Ministry of Health that all Malawians live within 8km of a health facility, and the construction of additional facilities should continue to prioritize those areas where people are furthest from care.

Limitations

These findings must be considered in light of several limitations. First, individual data does not indicate where vaccination services were rendered; children may have received immunization services from an alternate facility than the one most proximal to them (including via outreach, the locations of which were not assessed in the SPA). Thus, it would be inappropriate to suggest a causal relationship between the most proximal facility's immunization service availability and an individual child's immunization. Additionally, outreach services are widely used in this population, with more than 5000 fixed and mobile outreach clinics throughout the country;[39] the observed association of immunization coverage with distance to static clinics likely underestimates the true strength of association with distance to location where vaccination was actually received. Second, general service availability may not reflect actual service readiness and availability at the time of immunization receipt. Third, three planned sensitivity analyses were not possible: separately analyzing facilities offering within-facility and outreach services was not possible as 99% of children who were proximal to a vaccine-providing facility were proximal to one offering both in-facility and outreach services; examining specific vaccination availability was not possible because 99% of children who were proximal to a vaccine-providing facility were proximal to one offering all six examined vaccines; examining receipt of vaccines within vs after the first year was not possible because >99% of children who had vaccination dates available received all vaccines by age one (with the exception of measles vaccine, which had 93% receipt by age one). Fourth, while the most recent available surveys were used, at time of publication these data are now 6-9 years old; additional research using more recent data will add insight into current realities. Furthermore, there was a 2-3 year gap between the survey of facilities (2013-14) and survey of individuals (2015-16, children born October 2013-February 2015), and facilities may have closed, opened, changed service offerings, or had fluctuations in vaccine stock over that time frame. However, retroactive reporting of immunization reflects immunization receipt close to the time of facility survey, and provision of any vaccination services is likely to have remained constant over that time. The lack of association between vaccine stock and immunization coverage may be due in part to the asynchronous surveys. Finally, the use of buffer distances does not account for actual travel distance, geography, or time, nor does it account for seasonal differences in physical access (e.g. due to rains). Similarly, the use of DHS cluster centroids does not reflect the actual household geolocation. Statistically, this will bias findings toward the null as the buffer distance used will not be precise; any associations observed are likely underestimates of the true strength of association. Recent studies support the use of other methods such as theoretical catchment areas for more accurate facility linkage.[52] However, given the research question

For peer review only

and the variable size of administrative and catchment areas, buffer distance was considered appropriate for these analyses.

CONCLUSION

Findings from this study align with previous work demonstrating a significant association between immunization coverage and distance to vaccine-providing facilities,[16-19] and expand on this by utilizing a nationally-representative sample with a focus on children living in rural areas. Remote rural populations have been identified as a key target for improving immunization equity, and these findings reiterate the vulnerability of children residing far from static vaccination services. Efforts that target remote rural populations living far from health facilities, even using crude measures of identification such as straight-line distance from facilities, are warranted to ensure equitable vaccination coverage. These analyses also suggest that health facility-level data can and should be used for further analyses of inequalities in immunization.

For peer review only

Abbreviations

AOR: adjusted odds ratio; aRRR: adjusted relative risk ratio; BCG: *Bacillus Calmette-Guérin*; CHAM: Christian Health Association of Malawi; CI: confidence interval; DHS: Demographic and Health Survey; DTP: diphtheria, tetanus, pertussis vaccine; EPI: Expanded Programme on Immunization; GPS: global positioning system; HBV: hepatitis B virus; Hib: *Haemophilus influenzae* type b; KM: kilometer; NGO: non-governmental organization; OPV: oral polio vaccine; PCV13: pneumococcal conjugate vaccine 13-valent; SIA: supplementary immunization activity; SPA: Service Provision Assessment

Funding

Funding for this work was provided by Gavi, the Vaccine Alliance. The funding source had no role in the study design, analysis, interpretation of data, or decision to publish results.

Disclaimer

The authors alone are responsible for the views expressed in this article and they do not necessarily represent the views, decisions or policies of the institutions with which they are affiliated.

Data availability

Data for this study are publicly available upon request through the DHS Program. The data are owned by the Government of Malawi and are archived and managed by The DHS Program. Interested researchers must register at https://dhsprogram.com/data/ and can then request permission to download the SPA and DHS datasets.

Analytical code for results presented here is available upon reasonable request from the corresponding author.

Conflicts of interests

The authors have no conflicts of interest to disclose.

Contributors

AH led initial study conceptualization and provided oversight of analyses and writing. NJ generated specific hypotheses, led data analyses, and created initial manuscript draft. MC, MS, and BZ provided country-specific context, content, and feedback for interpretation of the data. CD-H and SS provided immunization context, content, and feedback for interpretation of the data. KK and AS provided health equity context, content, and feedback for interpretation of the data. All authors contributed to substantive review and revision of the final manuscript, provided final approval of the version to be published, and agree to be accountable for all aspects of the work.

For peer review only

REFERENCES

1. Keja K, Chan C, Hayden G, et al. Expanded programme on immunization. *World health statistics quarterly Rapport trimestriel de statistiques sanitaires mondiales* 1988;41(2):59-63.
2. State of inequality: childhood immunization. Geneva: World Health Organization, 2016.
3. Galles NC, Liu PY, Updike RL, et al. Measuring routine childhood vaccination coverage in 204 countries and territories, 1980–2019: a systematic analysis for the Global Burden of Disease Study 2020, Release 1. *The Lancet* 2021;398(10299):503-21.
4. Muhoza P, Danovaro-Holliday MC, Diallo MS, et al. Routine Vaccination Coverage - Worldwide, 2020. *Mmwr-Morbid Mortal W* 2021;70(43):1495-500.
5. Explorations of inequality: Childhood immunization. Geneva, Switzerland: World Health Organization, 2018.
6. Hosseinpoor AR, Bergen N, Schlotheuber A, et al. State of inequality in diphtheria-tetanus-pertussis immunisation coverage in low-income and middle-income countries: a multicountry study of household health surveys. *The Lancet Global Health* 2016;4(9):e617-e26.
7. Levine O, Lemango E, Befson J, et al. ERG Discussion Paper 08: Tackling inequities in immunization outcomes in remote rural contexts. 2018. https://sites.google.com/view/erg4immunisation/discussion-papers (accessed 09/21/21).
8. Chopra M, Bhutta Z, Blanc DC, et al. Addressing the persistent inequities in immunization coverage. *B World Health Organ* 2020;98(2):146-48. doi: 10.2471/Blt.19.241620
9. World Health Organization. Immunization Agenda 2030: a global strategy to leave no one behind. *Geneva: WHO* 2020
10. Lindstrand A, Cherian T, Chang-Blanc D, et al. The World of Immunization: Achievements, Challenges, and Strategic Vision for the Next Decade. *The Journal of infectious diseases* 2021;224(Supplement_4):S452-S67.
11. Gavi The Vaccine Alliance. Phase V (2021–2025) 2019 [updated 9 Jun 2021. Available from: https://www.gavi.org/our-alliance/strategy/phase-5-2021-2025.
12. Bangura JB, Xiao S, Qiu D, et al. Barriers to childhood immunization in sub-Saharan Africa: A systematic review. *BMC Public Health* 2020;20(1):1108. doi: 10.1186/s12889-020-09169-4 [published Online First: 2020/07/16]
13. Rahman M, Obaida-Nasrin S. Factors affecting acceptance of complete immunization coverage of children under five years in rural Bangladesh. *Salud Publica Mex* 2010;52(2):134-40. doi: 10.1590/s0036-36342010000200005
14. Adedokun ST, Uthman OA, Adekanmbi VT, et al. Incomplete childhood immunization in Nigeria: a multilevel analysis of individual and contextual factors. *BMC Public Health* 2017;17(1):236. doi: 10.1186/s12889-017-4137-7
15. Phillips DE, Dieleman JL, Lim SS, et al. Determinants of effective vaccine coverage in low and middle-income countries: a systematic review and interpretive synthesis. *Bmc Health Serv Res* 2017;17 doi: ARTN 681
16. Tefera YA, Wagner AL, Mekonen EB, et al. Predictors and Barriers to Full Vaccination among Children in Ethiopia. *Vaccines* 2018;6(2) doi: 10.3390/vaccines6020022
17. Miyahara R, Jasseh M, Gomez P, et al. Barriers to timely administration of birth dose vaccines in The Gambia, West Africa. *Vaccine* 2016;34(29):3335-41. doi: 10.1016/j.vaccine.2016.05.017 [published Online First: 2016/05/20]
18. Kiptoo E, Esilaba M, Kobia G, et al. Factors influencing low immunization coverage among children between 12-23 months in East Pokot, Baringo Country, Kenya. *Int J Vaccines* 2015;1(2):00012.
19. Sato R. Association between access to a health facility and continuum of vaccination behaviors among Nigerian children. *Hum Vacc Immunother* 2020;16(5):1215-20. doi: 10.1080/21645515.2019.1678360
20. Ministry of Health. Financial Sustainability Plan (FSP) for Expanded Programme on Immunization. Linongwe, Malawi, 2004.
21. Munthali AC. Determinants of vaccination coverage in Malawi: evidence from the demographic and health surveys. *Malawi Med J* 2007;19(2):79-82. doi: 10.4314/mmj.v19i2.10934 [published Online First: 2007/06/01]
22. Nyirenda L, Flikke R. Frontline Vaccinators and Immunisation Coverage in Malawi. *Forum Dev Stud* 2013;40(1):27-46. doi: 10.1080/08039410.2012.725676
23. Minetti A, Kagoli M, Katsulukuta A, et al. Lessons and challenges for measles control from unexpected large outbreak, Malawi. *Emerg Infect Dis* 2013;19(2):202-9. doi: 10.3201/eid1902.120301
24. Chinele J. Over a million children get protected against polio in Malawi. 2021 26 August 2021. https://www.gavi.org/vaccineswork/over-million-children-get-protected-against-polio-malawi (accessed 09/21/2021).

For peer review only

25. Kainga HW, Ssendagire S, Ssanyu JN, et al. Proportion of children aged 9-59 months reached by the 2017 measles supplementary immunization activity among the children with or without history of measles vaccination in Lilongwe district, Malawi. *PLoS One* 2021;16(1):e0243137. doi: 10.1371/journal.pone.0243137
26. National Statistical Office (NSO) [Malawi] and ICF. Malawi Demographic and Health Survey 2015-16. Zomba, Malawi, and Rockville, Maryland, USA.: NSO and ICF., 2017.
27. National Statistical Office (NSO) [Malawi] and ICF. Malawi Demographic and Health Survey 2015-16 [Dataset]. In: National Statistical Office (NSO) [Malawi] and ICF [Producers], ed.: ICF [Distributor], 2017.
28. 2018 Population and Housing Census Main Report. Zomba, Malawi: Malawi National Statistical Office, 2019.
29. Ministry of Health. 2014/15 ANNUAL REVIEW REPORT FOR THE HEALTH SECTOR. Linongwe, Malawi, 2015.
30. Okeibunor JC, Ogbuanu I, Blanche A, et al. Towards a Strategy for Reducing Missed Opportunities for Vaccination in Malawi: Implications of a Qualitative Health Facility Assessment. *J Immunol Sci* 2018;Suppl(7):46-54.
31. Ntenda PAM, Nkoka O, Nana AW, et al. Factors associated with completion of childhood immunization in Malawi: a multilevel analysis of the 2015-16 Malawi demographic and health survey. *Trans R Soc Trop Med Hyg* 2019 doi: 10.1093/trstmh/trz029
32. McGuire F, Kreif N, Smith PC. The effect of distance on maternal institutional delivery choice: Evidence from Malawi. *Health Econ* 2021;30(9):2144-67. doi: 10.1002/hec.4368
33. Leslie HH, Fink G, Nsona H, et al. Obstetric Facility Quality and Newborn Mortality in Malawi: A Cross-Sectional Study. *PLoS Med* 2016;13(10):e1002151. doi: 10.1371/journal.pmed.1002151
34. Peven K, Taylor C, Purssell E, et al. Distance to available services for newborns at facilities in Malawi: A secondary analysis of survey and health facility data. *PloS one* 2021;16(7):e0254083.
35. Mallick L, Benedict RK, Wang W. Facility readiness and counseling during antenatal care and the relationship with early breastfeeding in Haiti and Malawi. *BMC Pregnancy Childbirth* 2020;20(1):325. doi: 10.1186/s12884-020-02919-7
36. Benedict R, Wang W, Mallick L. Examining the Role of Health Facilities in Supporting Iron Folic Acid Supplementation Adherence Among Women in Malawi (OR25-03-19). *Current developments in nutrition* 2019;3(Supplement_1):nzz051. OR25-03-19.
37. Digitale J, Psaki S, Soler-Hampejsek E, et al. Correlates of contraceptive use and health facility choice among young women in Malawi. *The ANNALS of the American Academy of Political and Social Science* 2017;669(1):93-124.
38. Ministry of Health - MoH/Malawi and ICF International. Malawi Service Provision Asessment (MSPA) 2013-14. Lilongwe, Malawi: MOH/Malawi and ICF International, 2014.
39. EPI Comprehensive Multi-Year Plan 2016-2020. 2015. https://extranet.who.int/countryplanningcycles/sites/default/files/planning_cycle_repository/malawi/malawi_cmyp_2016-2020.pdf (accessed 09/21/2021).
40. Burgert CR, Prosnitz D. Linking DHS Household and SPA Facility Surveys: Data Considerations and Geospatial Methods. DHS Spatial Analysis Reports No 10. Rockville, Maryland, USA: ICF, 2014.
41. Fish TD, Janocha B, Dontamsetti T, et al. GEOSPATIAL COVARIATES: PROXIES FOR MAPPING URBAN-RELATED INDICATORS. DHS SPATIAL ANALYSIS REPORTS 19. Rockville, Maryland, USA: ICF, 2020.
42. Wiysonge CS, Uthman OA, Ndumbe PM, et al. Individual and contextual factors associated with low childhood immunisation coverage in sub-Saharan Africa: a multilevel analysis. *PloS one* 2012;7(5):e37905.
43. Ozawa S, Yemeke TT, Evans DR, et al. Defining hard-to-reach populations for vaccination. *Vaccine* 2019;37(37):5525-34. doi: 10.1016/j.vaccine.2019.06.081
44. Mandomando I, Mumba M, Biey JNM, et al. Implementation of the World Health Organization recommendation on the use of rotavirus vaccine without age restriction by African countries. *Vaccine* 2021;39(23):3111-19. doi: 10.1016/j.vaccine.2021.03.021
45. Makwero MT. Delivery of primary health care in Malawi. *Afr J Prim Health Ca* 2018;10(1) doi: ARTN a1799
46. Olorunsaiye CZ, Yusuf KK, Reinhart K, et al. COVID-19 and Child Vaccination: A Systematic Approach to Closing the Immunization Gap. *International Journal of Maternal and Child Health and AIDS* 2020;9(3):381.
47. Shet A, Carr K, Danovaro-Holliday MC, et al. Impact of the SARS-CoV-2 pandemic on routine immunisation services: evidence of disruption and recovery from 170 countries and territories. *Lancet Glob Health* 2021 doi: 10.1016/S2214-109X(21)00512-X
48. Dixit SM, Sarr M, Gueye DM, et al. Addressing disruptions in childhood routine immunisation services during the COVID-19 pandemic: perspectives from Nepal, Senegal and Liberia. *Bmj Glob Health* 2021;6(7) doi: ARTN e005031

For peer review only

49. Chinele J. Tackling Malawi's fears of routine immunisation: "Children should still be immunised amid the pandemic": Gavi: The Vaccine Alliance; 2021 [updated 24 June 2021. Available from: https://www.gavi.org/vaccineswork/tackling-malawis-fears-routine-immunisation-children-should-still-be-immunised-amid.
50. World Health Organization, UNICEF. Malawi: WHO and UNICEF estimates of immunization coverage: 2020 revision, 2021.
51. World Health Organization. Leave no one behind : guidance for planning and implementing catch-up vaccination, 2021.
52. Peters MA, Mohan D, Naphini P, et al. Linking household surveys and facility assessments: a comparison of geospatial methods using nationally representative data from Malawi. *Population health metrics* 2020;18(1):1-11.

For peer review only

Appendix Table 1. Additional study sample characteristics, children aged 12-23 months in rural areas, Malawi DHS 2015-16.

	Unweighted N	Weighted %
Total	*2740*	*100.0%*
**Individual child indicators**		
Sex of child		
Male	1362	49.1%
Female	1378	50.9%
Birth order		
1st	673	25.3%
2nd	522	18.9%
3rd	453	16.1%
4th	375	13.2%
5th or more	717	26.5%
**Maternal/household indicators**		
Mother's age (Weighted Mean / SD)	*27.2*	*6.6*
Mother's education		
None	331	13.2%
Primary	1963	71.7%
Secondary or higher	446	15.2%
Household wealth index quintile*		
Poorest	769	29.9%
Poorer	678	24.8%
Middle	601	22.3%
Richer	464	16.1%
Richest	228	7.0%
Region of Malawi		
Northern	508	12.0%
Central	959	42.0%
Southern	1273	46.0%

For peer review only

Appendix Table 2. Unadjusted and adjusted comparisons of immunization rates among children in rural areas aged 12-23 months by presence of vaccine-providing facility in 8km radius, Malawi 2013-14.

	Unadjusted rates			Unadjusted logistic regression		Adjusted logistic regression	
	No vaccine-providing facility proximal	Vaccine-providing facility proximal	p-value	OR[1]	95% CI	AOR[1,2]	95% CI
Specific immunization dose coverage							
BCG	96.4%	97.8%	0.22	1.59	[0.71,3.58]	1.54	[0.70,3.39]
Rotavirus 2 doses	87.0%	91.9%	0.02	1.66*	[1.03,2.65]	1.53	[0.95,2.45]
OPV 3 doses	80.4%	81.3%	0.75	1.14	[0.78,1.67]	1.14	[0.80,1.63]
Pentavalent 3 doses	90.7%	93.3%	0.25	1.55	[0.95,2.83]	1.44	[0.81,2.56]
Pneumococcal 3 doses	87.2%	89.4%	0.54	1.33	[0.67,2.64]	1.20	[0.64,2.26]
Measles 1+ dose	86.0%	92.0%	0.03	2.04*	[1.16,3.60]	1.88*	[1.06,3.33]
Coverage of group of immunizations							
All basic vaccines[3]	69.1%	76.6%	0.08	1.57*	[1.02,2.43]	1.54*	[1.02,2.32]
All recommended vaccines[4]	36.7%	53.2%	<0.001	1.89**	[1.28,2.81]	1.97***	[1.35,2.89]
Negative immunization outcomes							
Pentavalent zero dose	5.4%	2.3%	0.03	0.37*	[0.16,0.88]	0.38*	[0.18,0.82]
Pentavalent dropout[5]	4.1%	4.6%	0.75	1.02	[0.51,2.01]	1.10	[0.54,2.24]

[1]Reference is children not proximal to a vaccine-providing facility
[2]Models also control for household wealth, mother's education, mother's age, child sex, child birth order, region of country
[3]Defined as BCG 1 dose, OPV 3 doses, DTP/HBV/Hib (pentavalent) 3 doses, measles 1 dose
[4]Basic + OPV birth dose + rotavirus 2 doses + PCV13 3 doses
[5]Dropout denominator is children with at least 1 dose of pentavalent
* p<0.05, ** p<0.01, *** p<0.001

For peer review only

Appendix Table 3a. Logistic regression models of individual-level vaccination among children 12-23 months living in rural areas who are within 5km of a facility providing vaccination. Only facility-characteristic coefficients reported.[1]

	Specific immunization dose coverage											
	**BCG**		**Rotavirus 2 dose**		**Polio 3 dose**		**Pentavalent 3 dose**		**Pneumococcal 3 dose**		**Measles 1+ dose**	
	AOR	95% CI	AOR	95% CI	AOR	95% CI	AOR	95% CI	AOR	95% CI	AOR	95% CI
Facility type offering vaccination [highest available if >1]												
Hospital	*Ref*	*Ref*	*Ref*	*Ref*	*Ref*	*Ref*	*Ref*	*Ref*	*Ref*	*Ref*	*Ref*	*Ref*
Health centre	0.92	[0.34,2.49]	1.03	[0.54,1.95]	1.07	[0.72,1.58]	0.89	[0.48,1.64]	0.69	[0.40,1.20]	1.17	[0.51,2.64]
Health post/clinic/dispensary	1.88	[0.42,8.48]	0.93	[0.38,2.28]	1.94	[0.97,3.86]	0.84	[0.33,2.16]	0.87	[0.34,2.24]	0.79	[0.27,2.34]
Managing authority of facility/ies offering vaccination												
Government												
No	*Ref*	*Ref*	*Ref*	*Ref*	*Ref*	*Ref*	*Ref*	*Ref*	*Ref*	*Ref*	*Ref*	*Ref*
Yes	2.06	[0.51,8.36]	1.65	[0.89,3.07]	2.32**	[1.37,3.93]	1.76	[0.91,3.42]	1.18	[0.64,2.15]	1.34	[0.70,2.55]
CHAM												
No	*Ref*	*Ref*	*Ref*	*Ref*	*Ref*	*Ref*	*Ref*	*Ref*	*Ref*	*Ref*	*Ref*	*Ref*
Yes	1.71	[0.44,6.67]	1.54	[0.80,2.97]	2.47***	[1.46,4.18]	2.04*	[1.06,3.93]	1.05	[0.60,1.86]	1.39	[0.63,3.07]
Private for-profit												
No	*Ref*	*Ref*	*Ref*	*Ref*	*Ref*	*Ref*	*Ref*	*Ref*	*Ref*	*Ref*	*Ref*	*Ref*
Yes	2.85	[0.31,26.02]	9.83*	[1.20,80.47]	0.82	[0.38,1.79]	5.36	[0.95,30.14]	1.96	[0.33,11.49]	1.57	[0.32,7.73]
NGO												
No	*Ref*	*Ref*	*Ref*	*Ref*	*Ref*	*Ref*	*Ref*	*Ref*	*Ref*	*Ref*	*Ref*	*Ref*
Yes	0.53	[0.13,2.18]	1.42	[0.42,4.81]	0.57	[0.26,1.28]	2.14	[0.54,8.44]	0.76	[0.27,2.14]	1.59	[0.46,5.48]

[1]Models also control for household wealth, education, mother's age, child sex, child birth order, region of country
* p<0.05, ** p<0.01, *** p<0.001

For peer review only

Appendix Table 3b. Logistic regression models of individual-level vaccination among children 12-23 months living in rural areas who are within 5km of a facility providing vaccination. Only facility-characteristic coefficients reported.[1]

	Coverage of group of immunizations				*Negative immunization outcomes*			
	All basic vaccines[2]		All recommended vaccines[3]		Pentavalent 0 dose		Pentavalent dropout	
	AOR	95% CI	AOR	95% CI	AOR	95% CI	AOR	95% CI
Facility type offering vaccination [highest available if >1]								
Hospital	*Ref*	*Ref*	*Ref*	*Ref*	*Ref*	*Ref*	*Ref*	*Ref*
Health centre	1.11	[0.75,1.66]	0.79	[0.55,1.14]	1.46	[0.42,5.12]	0.95	[0.49,1.86]
Health post/clinic/dispensary	1.45	[0.75,2.81]	0.93	[0.56,1.54]	0.20	[0.03,1.28]	1.53	[0.57,4.09]
Managing authority of facility/ies offering vaccination								
Government								
No	*Ref*	*Ref*	*Ref*	*Ref*	*Ref*	*Ref*	*Ref*	*Ref*
Yes	1.82*	[1.15,2.88]	1.94**	[1.24,3.03]	0.08***	[0.02,0.33]	0.97	[0.44,2.11]
CHAM								
No	*Ref*	*Ref*	*Ref*	*Ref*	*Ref*	*Ref*	*Ref*	*Ref*
Yes	2.29***	[1.44,3.63]	1.33	[0.86,2.07]	0.11**	[0.02,0.50]	0.64	[0.31,1.32]
Private for-profit								
No	*Ref*	*Ref*	*Ref*	*Ref*	--	--	*Ref*	*Ref*
Yes	0.84	[0.39,1.78]	1.04	[0.48,2.25]	--	--	0.26	[0.04,1.54]
NGO								
No	*Ref*	*Ref*	*Ref*	*Ref*	--	--	*Ref*	*Ref*
Yes	0.60	[0.28,1.28]	0.62	[0.35,1.10]	--	--	0.60	[0.15,2.32]

[1]Models also control for household wealth, education, mother's age, child sex, child birth order, region of country
[2]Defined as BCG 1 dose, OPV 3 dose, DTP/HBV/Hib (pentavalent) 3 dose, measles 1 dose
[3]Basic + OPV birth dose + rotavirus 2 doses + PCV13 3 doses
* p<0.05, ** p<0.01, *** p<0.001

For peer review only

STROBE Statement—Checklist of items that should be included in reports of ***cross-sectional studies***

	Item No	Recommendation	Page No
**Title and abstract**	1	(*a*) Indicate the study's design with a commonly used term in the title or the abstract	1
		(*b*) Provide in the abstract an informative and balanced summary of what was done and what was found	2
**Introduction**			
Background/rationale	2	Explain the scientific background and rationale for the investigation being reported	4-5
Objectives	3	State specific objectives, including any prespecified hypotheses	5
**Methods**			
Study design	4	Present key elements of study design early in the paper	5-6
Setting	5	Describe the setting, locations, and relevant dates, including periods of recruitment, exposure, follow-up, and data collection	5-6
Participants	6	(*a*) Give the eligibility criteria, and the sources and methods of selection of participants	6
Variables	7	Clearly define all outcomes, exposures, predictors, potential confounders, and effect modifiers. Give diagnostic criteria, if applicable	7
Data sources/ measurement	8*	For each variable of interest, give sources of data and details of methods of assessment (measurement). Describe comparability of assessment methods if there is more than one group	7
Bias	9	Describe any efforts to address potential sources of bias	8, 15
Study size	10	Explain how the study size was arrived at	6, 9
Quantitative variables	11	Explain how quantitative variables were handled in the analyses. If applicable, describe which groupings were chosen and why	7
Statistical methods	12	(*a*) Describe all statistical methods, including those used to control for confounding	7-8
		(*b*) Describe any methods used to examine subgroups and interactions	8
		(*c*) Explain how missing data were addressed	7
		(*d*) If applicable, describe analytical methods taking account of sampling strategy	8
		(*e*) Describe any sensitivity analyses	8
**Results**			
Participants	13*	(a) Report numbers of individuals at each stage of study—eg numbers potentially eligible, examined for eligibility, confirmed eligible, included in the study, completing follow-up, and analysed	9
		(b) Give reasons for non-participation at each stage	N/A
		(c) Consider use of a flow diagram	N/A
Descriptive data	14*	(a) Give characteristics of study participants (eg demographic, clinical, social) and information on exposures and potential confounders	9-10
		(b) Indicate number of participants with missing data for each variable of interest	7
Outcome data	15*	Report numbers of outcome events or summary measures	8-9
Main results	16	(*a*) Give unadjusted estimates and, if applicable, confounder-adjusted estimates and their precision (eg, 95% confidence interval). Make clear which confounders were adjusted for and why they were included	10-12

For peer review only - http://bmjopen.bmj.com/site/about/guidelines.xhtml

		(*b*) Report category boundaries when continuous variables were categorized	7
		(*c*) If relevant, consider translating estimates of relative risk into absolute risk for a meaningful time period	N/A
Other analyses	17	Report other analyses done—eg analyses of subgroups and interactions, and sensitivity analyses	13
**Discussion**			
Key results	18	Summarise key results with reference to study objectives	3-14
Limitations	19	Discuss limitations of the study, taking into account sources of potential bias or imprecision. Discuss both direction and magnitude of any potential bias	15
Interpretation	20	Give a cautious overall interpretation of results considering objectives, limitations, multiplicity of analyses, results from similar studies, and other relevant evidence	14-15
Generalisability	21	Discuss the generalisability (external validity) of the study results	14-15
**Other information**			
Funding	22	Give the source of funding and the role of the funders for the present study and, if applicable, for the original study on which the present article is based	13

*Give information separately for exposed and unexposed groups.

Note: An Explanation and Elaboration article discusses each checklist item and gives methodological background and published examples of transparent reporting. The STROBE checklist is best used in conjunction with this article (freely available on the Web sites of PLoS Medicine at http://www.plosmedicine.org/, Annals of Internal Medicine at http://www.annals.org/, and Epidemiology at http://www.epidem.com/). Information on the STROBE Initiative is available at www.strobe-statement.org.

For peer review only

For peer review only - http://bmjopen.bmj.com/site/about/guidelines.xhtml
